# Nobiletin affects circadian rhythms and oncogenic characteristics in a cell-dependent manner

**DOI:** 10.1371/journal.pone.0236315

**Published:** 2020-07-24

**Authors:** Sujeewa S. Lellupitiyage Don, Kelly L. Robertson, Hui-Hsien Lin, Caroline Labriola, Mary E. Harrington, Stephanie R. Taylor, Michelle E. Farkas

**Affiliations:** 1 Department of Chemistry, University of Massachusetts Amherst, Amherst, MA, United States of America; 2 Department of Biochemistry & Molecular Biology, University of Massachusetts Amherst, Amherst, MA, United States of America; 3 Department of Psychology, Smith College, Northampton, MA, United States of America; 4 Department of Computer Science, Colby College, Waterville, ME, United States of America; McGill University, CANADA

## Abstract

The natural product nobiletin is a small molecule, widely studied with regard to its therapeutic effects, including in cancer cell lines and tumors. Recently, nobiletin has also been shown to affect circadian rhythms via their enhancement, resulting in protection against metabolic syndrome. We hypothesized that nobiletin’s anti-oncogenic effects, such as prevention of cell migration and formation of anchorage independent colonies, are correspondingly accompanied by modulation of circadian rhythms. Concurrently, we wished to determine whether the circadian and anti-oncogenic effects of nobiletin differed across cancer cell lines. In this study, we assessed nobiletin’s circadian and therapeutic characteristics to ascertain whether these effects depend on cell line, which here also varied in terms of baseline circadian rhythmicity. Three cell culture models where nobiletin’s effects on cell proliferation and migration have been studied previously were evaluated: U2OS (bone osteosarcoma), which possesses robust circadian rhythms; MCF7 (breast adenocarcinoma), which has weak circadian rhythms; and MDA-MB-231 (breast adenocarcinoma), which is arrhythmic. We found that circadian, migration, and proliferative effects following nobiletin treatment were subtle in the U2OS and MCF7 cells. On the other hand, changes were clear in MDA-MB-231s, where nobiletin rescued rhythmicity and substantially reduced oncogenic features, specifically two-dimensional cell motility and anchorage-independent growth. Based on these results and those previously described, we posit that the effects of nobiletin are indeed cell-type dependent, and that a positive correlation may exist between nobiletin’s circadian and therapeutic effects.

## Introduction

Nobiletin is a polymethoxylated flavone present in the peels of citrus fruits [1]. It has been reported to yield therapeutic effects in a variety of disease models, including those of neurological, inflammatory, cardiac, and metabolic diseases, in addition to cancer cell lines and tumors. Recently, it has also been shown to affect circadian oscillations [2,3]. We are interested in concomitant studies of nobiletin’s therapeutic and circadian effects. Previous studies have shown that nobiletin can improve memory in Alzheimer’s [4] and motor deficits in Parkinson’s disease models [5], and can result in anti-depressant-like effects (i.e. reduced immobility times in forced swimming and tail suspension tests in mice) [6]. It can also ameliorate adiposity, hyperlipidemia, hyperglycemia, and insulin resistance [7], attenuate lipid accumulation [8], protect against metabolic syndrome [2], reduce excessive inflammatory responses and restore epithelial barrier function in colitis [9], and protect against acute pancreatitis [10]. For heart diseases, nobiletin results in neuroprotection of cerebral ischemia-reperfusion [11], lowers serum low-density lipoprotein (LDL)/very low-density lipoprotein (VLDL) cholesterol [12], and has been shown to ameliorate cardiac dysfunction [13]. In mouse models, nobiletin has been shown to affect aging processes, including inhibition of bone resorption and maintenance of bone mass [14]. On these accounts, nobiletin has the potential to serve as a therapeutic entity against numerous conditions.

Treatment with nobiletin has resulted in anti-oncogenic effects in multiple cancer cell lines and tumor types by affecting major pathways including protein kinase B (AKT), mitogen-activated protein kinase (MAPK), transforming growth factor beta-1 (TGF-β1)/SMAD3, extracellular-signal-regulated kinase (ERK), and c-Jun N-terminal kinase (JNK). In studies where it was shown to affect the AKT pathway, nobiletin was found to decrease tumor viability, weight, and volume in A2780/CP70 ovarian cancer xenograft models [15]. It also reduced cell adhesion, invasion, and migration in the highly metastatic AGS gastric adenocarcinoma cell line [16], suppressed viability of PC-3 and DU-145 prostate cancer cell lines [17], and inhibited ACHN and Caki-2 renal carcinoma cell proliferation by cell cycle arrest in G0/G1 phase [18]. Also, nobiletin reduced proliferation and induced apoptosis in HL-60 human acute myeloid leukemia cells [19], diminished proliferation and migration in U87 glioma cells [20], and decreased cell proliferation in the low tumor-grade MCF7 cell line via G1 cell cycle block [21]. It may induce apoptosis and inhibit cell migration via MAPK and/or MAPK/AKT related pathways [22,23]. Similar anti-proliferative effects have been observed in high tumor grade MDA-MB-231 [23] and MDA-MB-468 human breast cancer cells following treatment with nobiletin [24]. Nobiletin has also been shown to inhibit growth of metastatic nodules in the lungs of mice via the TGF-β1/SMAD3 pathway [25] and decrease cell migration, angiogenesis, tumor formation, and progression in the bone osteosarcoma cell line U2OS via ERK and JNK pathways [26]. Taken together, nobiletin is effective against cell lines and tumors representing various cancer types, and exerts its effects via various pathways.

While identification of small molecules that can modulate circadian rhythms has garnered significant interest [27], it has recently been shown that many existing therapeutic compounds also affect circadian rhythms [28]. In a similar fashion, nobiletin, which has been known for decades to elicit various beneficial health effects, has only recently been shown to alter circadian rhythms [2,3]. Circadian rhythms are 24-hour rhythmic activity cycles. At the molecular level, they are regulated by circadian locomoter output cycles kaput (CLOCK), brain and muscle ARNT-Like 1 (BMAL1), period (PER), and cryptochrome (CRY) proteins, which exist in a negative feed-back loop [29]. CLOCK and BMAL1 are positive components that result in gene expression, including of the negative regulators PER1, PER2, PER3, CRY1, and CRY2. Nobiletin has been shown to activate a critical component of a secondary loop, retinoic acid-related orphan receptor (ROR) [2], which binds to the *Bmal1* promoter, activating its transcription [29]. While other targets may exist, and at least two have been computationally predicted [23,30], they have yet to be determined. Two prior studies have assessed nobiletin’s circadian effects. In the first, mouse embryonic fibroblasts (MEFs) from heterozygous *Clock* knockout mice and mouse ear fibroblasts from *PER2∷LucSV* reporter mice showed increased *PER2*:*Luc* amplitudes, which were confirmed to be the result of increased ROR activity [2]. In a second study, nobiletin similarly increased amplitudes but also lengthened the periods of *PER2*::*Luc* in MEFs, while inducing phase delays in liver slices from *PER2*::*Luc* knock-in mice [3]. However, despite nobiletin’s therapeutic applications, to our knowledge, its simultaneous effects on circadian rhythms have been addressed only in a single study [2]. Therefore, here we assess nobiletin’s circadian and therapeutic effects in parallel, under the same treatment conditions and models.

Given the substantial evidence of nobiletin’s efficacy in reducing cell migration and proliferation in cancer cell lines and tumors, we considered it important to evaluate its circadian effects in these systems. In this study, we used three cancer cell lines, each of which nobiletin has shown activity in: U2OS, MCF7, and MDA-MB-231. Based on previous work, U2OS is a cell line with robust circadian rhythms [31], the low-grade breast cancer cell line MCF7 has low amplitude oscillations, and the high tumor grade breast cancer cell line MDA-MB-231 is arrhythmic [32]. We were particularly interested in determining whether the extent of oscillation robustness and nobiletin-influenced circadian changes could be correlated with anti-oncogenic effects. To assess this, we dosed the cells with two concentrations of nobiletin and evaluated how circadian rhythms are affected using real-time luminometry. We evaluated the oncogenic features of cell migration and anchorage-independent clonal expansion using wound healing and colony formation assays, respectively. Upon nobiletin treatment, we found subtle circadian alterations in U2OS and MCF7 cells, but strikingly observed enhanced rhythmicity in otherwise arrhythmic MDA-MB-231 cells [32]. Under the same conditions, oncogenic features in U2OS cells were disparately affected, with motility reduced, but increased colony areas observed. In MCF7 cells, cell migration was not altered, but at the highest concentration, colony areas decreased. The most significant changes were observed in nobiletin treated MDA-MB-231 cells, which possessed reductions in both cell migration and colony area.

## Materials and methods

### Cell culture

U2OS [33] cells were obtained from Prof. Patricia Wadsworth (Biology, UMass Amherst), MCF7 [34] cells were obtained from Prof. D. Joseph Jerry (Veterinary and Animal Sciences, UMass Amherst), and MDA-MB-231 [35] cells were obtained from Prof. Shelly Peyton (Chemical Engineering, UMass Amherst). MCF7 cells were characterized for expression of estrogen receptor and its signaling. MDA-MB-231s were evaluated for karyotype and subtype confirmation via RNAseq prior to transfer. Both cell lines were tested for mycoplasma via PCR detection. U2OS cells were maintained in Dulbecco’s Modified Eagle Medium (DMEM; Gibco), with 10% Fetal Bovine Serum (FBS; Corning), 1% L-Glutamine (L-glut; Gibco), 1% penicillin-streptomycin (Gibco), 1% Non-Essential Amino Acids (HyClone), and 1% Sodium Pyruvate (Gibco) [36]. MCF7 and MDA-MB-231 cells were maintained in DMEM (Gibco), supplemented with 10% FBS (Corning), 1% penicillin-streptomycin (Gibco), and 1% L-glut (Gibco) [32]. All cells were incubated at 37°C under 5% CO_2_ atmosphere, unless otherwise noted.

### Lentiviral transductions

The generation of *Bmal1*:*luc* and *Per2*:*luc* plasmids [37] and their subsequent stable transfection into U2OS [36], MCF7, and MDA-MB-231 [32] cells have been described previously.

### Nobiletin treatments

Nobiletin (Acros Organics, catalog number AC463522500, lot number A0390078) was prepared in 100% dimethyl-sulfoxide (DMSO; Sigma-Aldrich) at a concentration of 191 mM and stored at -20°C in single use aliquots. When dosing cells, the nobiletin stock was serially diluted in DMSO to achieve the desired concentration while maintaining a final, constant DMSO concentration of 0.2% (including vehicle controls) in media. Media, prepared according to experiment type, containing DMSO/nobiletin was added to synchronized cells (described below) and left in the dish for the duration of each experiment.

### Cell synchronization

Cells were seeded in 35 mm culture dishes in 2 mL at a density of 2 x 10^5^ cells/mL and incubated for 2–4 days. For luminometry, synchronization was performed when U2OS and MDA-MB-231 cells were confluent, while MCF7 cells were approximately 90% confluent. For RT-PCR and cell viability experiments all cells were confluent at the initiation of the experiments. Synchronization methods differed by cell line, and were based on previously reported protocols [32,38–43] that were optimized in our laboratory to obtain the best oscillations possible. U2OS growth media was then aspirated and 100 nM dexamethasone-containing culture media added for 2 h to synchronize the cells as in prior studies [28,44,45]. U2OS cells were not starved before synchronization. For MCF7, cells were washed with phosphate buffered saline (PBS) and synchronized by subjecting them to starvation conditions (DMEM with 1% L-glut) for 18 h followed by either treatment with 100 nM dexamethasone-containing DMEM with 1% L-glut (for luminometry) or 2 h serum shock in 1:1 FBS and DMEM with 1% L-glut (for wound healing and colony formation assays). The concentration and duration of dexamethasone treatment used here are not expected to synchronize the cell cycle, as shown previously [46]. For MDA-MB-231, cells were washed with PBS and synchronized via 18 h starvation in DMEM with 1% L-glut followed by 2 h serum shock in 1:1 FBS and DMEM with 1% L-glut. Cells were then treated according to experiment-specific procedures, described below.

### Cell viability assay

U2OS, MCF7, and MDA-MB-231 cells were seeded in a 24 well plate in 0.5 mL at a density of 2 x 10^5^ cells/mL, and grown to confluence. Depending on sample, cells were not treated, or treated with DMSO vehicle (to 0.2%), 5 μM nobiletin or 50 μM nobiletin (both with 0.2% DMSO). Cells were incubated for 24 h at 37°C in 5% CO_2_. Then the culture media was replaced with 10% Alamar blue reagent (Invitrogen) and incubated for 1 h under the same conditions. The Alamar blue reagent was then transferred to a black 96 well plate (Costar), and samples excited at 540 nm and emission detected at 590 nm using a Synergy H1 microplate reader.

### RNA isolation, cDNA conversion, and RT-PCR

U2OS, MCF7, and MDA-MB-231 cells were seeded in a 24 well plate in 0.5 mL at a density of 2 x 10^5^ cells/mL and grown for 2–4 days. Depending on sample, cells were not treated, or treated with DMSO vehicle (to 0.2%), 5 μM nobiletin or 50 μM nobiletin (both with 0.2% DMSO). Cells were then incubated for 24 h. After, the RNA was harvested as described previously [32]. Briefly, RNA was harvested from cells using a PureLink RNA Mini Kit (Ambion) according to the manufacturer’s instructions. RNA was reverse transcribed to cDNA using 50 μM random hexamers, 10 mM dNTPs, 40 U/μL RNaseOut, and 200 U/μL SuperScript IV Reverse Transcriptase, 100 mM DTT, and 5x SSIV buffer (Thermo Fisher Scientific). RT-PCR was performed in 96-well plates. Each reaction consisted of 100 ng cDNA, 10 μL iTaq universal SYBR Green Supermix (Biorad), 4 μM of each forward and reverse primer (Integrated DNA Technologies), and RNAse-free water to 20 μL. The primers used are *GAPDH* forward (5'- CTT CTT TTG CGT CGC CAG CC-3'), reverse (5'- ATT CCG TTG ACT CCG ACC TTC-3'); *Bmal1* forward (5’- CTA CGC TAG AGG GCT TCC TG-3’), reverse (5’- CTT TTC AGG CGG TCA GCT TC-3’); *Per2* forward (5’- TGT CCC AGG TGG AGA GTG GT-3’), reverse (5’- TGT CAC CGC AGT TCA AAC GAG-3’). The samples were centrifuged and analyzed via CFX Connect real-time system (Biorad) using an initial denaturation at 95°C for 3 min, followed by 40 cycles of 95°C denaturation for 10 s, and 60°C annealing/extension for 30 s. Relative *Bmal1* and *Per2* expression were determined by comparing *C*_*t*_ values of *Bmal1* and *Per2* to *GAPDH* (control) via 2^ΔΔCt^ method [47]. Three biological replicates and three technical replicates per biological replicate were analyzed for each condition.

### Bioluminescence recording and analysis

Following synchronization as described above, media was replaced with bioluminescence recording media. For U2OS cells, bioluminescence recording media was prepared by dissolving powdered DMEM (Sigma-Aldrich) in Millipore-purified water (18.2 MΩ.cm resistance) to give a final concentration of 0.01125 g/mL. This solution was sterile filtered using a 0.2 μm filter (Thermo Fisher) and used to prepare recording media containing the following additives (final concentrations are indicated here): 4mM sodium bicarbonate (Gibco), 5% FBS (Corning), 1% 4-(2-hydroxyethyl)-1-piperazineethanesulfonic acid (HEPES; HyClone), 0.25% penicillin-streptomycin (Gibco), and 150 μg/mL D-luciferin (Pierce). For MCF7 and MDA-MB-231 cells, recording media was prepared by dissolving powdered DMEM (Sigma-Aldrich) in Millipore water to give a final concentration of 0.0135 g/mL. This solution was sterile filtered using a 0.2 μm filter (Thermo Fisher) and used to prepare recording media containing the following additives (final concentrations are indicated here): 1% sodium pyruvate (Thermo Scientific), 5% FBS, 1% HEPES, 1% penicillin-streptomycin, and 150 μg/mL D-luciferin (Pierce). Dishes were sealed with 40 mm sterile cover glass using silicone vacuum grease and subjected to monitoring using a LumiCycle 32 System (Actimetrics) at 36.5°C for 5–7 days.

Each bioluminescence recording was pre-processed to remove an initial 12 h transient and spikes. Recordings from rhythmic cell lines (U2OS and MCF7) were then de-trended and circadian parameters estimated. Time-series were de-trended by removing the 24 h moving average (which excludes an additional 12 hours of data) and smoothed using the 3 h running average method. Circadian amplitude, period, and damping rate of each time-series were estimated with two approaches. The first was to fit the first three cycles (t = 24 h to t = 96 h) to a damped cosine curve with a nonlinear least squares method, using R (www.r-project.org) code adapted from Hirota et al [48]. The second approach was to identify the time and magnitude of each phase marker (peak, trough, mean-crossing on the rise, mean-crossing on the fall) of the first three cycles (custom Matlab script). Each phase marker was used to estimate a period (e.g. average peak-to-peak measurements led to one estimate and trough-to-trough to a second), providing four additional period estimates. The amplitude was estimated by adding the magnitude of the first trough to the magnitude of the first peak. To capture damping, the ratio of the amplitude of the second cycle to that of the first cycle was subtracted from unity.

### Wound healing assay

U2OS, MCF7, and MDA-MB231 cells were seeded in 0.5 mL at a density of 2 x 10^5^ cells/mL (U2OS) and ~4 x 10^5^ cells/mL (MCF7, MDA-MB-231), in 24-well plates and incubated until 100% confluence was reached (2–4 days). Cells were synchronized as described above. Wounds were generated using a 1 mL micropipette tip. Culture media was then removed, cells were washed with PBS, and 500 μL of new culture media containing indicated treatments was added into each well. Images were taken immediately following for the first time point (T = 0) and then every 2 h for 24 h via Biotek Cytation 3 cell imaging multi-mode plate reader. Wound closure (%) was determined by normalizing wound area quantified at each time point via macros adapted from MRI wound healing tool (http://dev.mri.cnrs.fr/projects/imagej-macros/wiki/Wound_Healing_Tool) to T = 0 via ImageJ.

### Colony formation assay

Cells were suspended in agarose and incubated until colonies formed, similarly to a previously described procedure [37]. Briefly, 3% 2-Hydroxyethyl Agarose (Sigma) was prepared and stored in a water bath at 45°C. Cell culture media at 37°C was used to dilute agarose to 0.6%, and 500 μL of the resulting solution was added to each well of a 24-well plate (Nuclon), which was incubated at 4°C until the agarose solidified to produce the first layer. The agarose solution was also added to the suspended cells (U2OS: 1 x 10^4^ cells/mL; MCF7 and MDA-MB-231 3.5 x 10^3^ cells/mL) in warm culture media to 0.3%, followed by addition of nobiletin. 500 μL of the drug-cell-agarose solution was dispensed per well and incubated at 4°C for 30 min until the second agarose layer solidified. Then plates were transferred and incubated at 37°C under 5% CO_2_. A feeder layer of 0.3% agarose in culture media containing nobiletin at designated concentrations (in 500 μL) was added to each well once every 7 days for 4 weeks. When imaging colonies, 20% methanol (Fisher Scientific) was added to wells, and plates were incubated on a shaker at rt for 1 h. Eight images were taken per well using a Biotek Citation 3 multi-mode cell imaging reader. Each condition was performed in three biological replicates. Images were stitched and colony numbers/sizes were analyzed using ImageJ software [49].

### Statistical analysis

To determine whether each bioluminescence time-series was rhythmic, we applied a fast fourier transform (FFT)-based statistical test designed for circadian bioluminescence recordings [50]. The null hypothesis was that the time-series exhibits characteristics of Brownian motion, meaning that the power spectrum is a linear function of the reciprocal of the frequency squared. The null hypothesis was rejected if the circadian peak of the power spectral density is above the regression line in log-log space (significance determined by a one-tailed Student’s t-test). Before applying the test, an exponential (for results in main text) or a quadratic (for supplementary results) trend was removed.

To compare circadian parameters between doses, a one-tailed randomization test for difference in means was used (http://www.lock5stat.com/StatKey/index.html). This test was chosen because a t-test was inappropriate (as it requires that each sample size is either large or normally distributed). In our case, N = 12 for each condition and not all were normally distributed. For each cell line, the test was performed for pairs of doses (e.g. non-treated to 5 μM nobiletin). The reference test statistic was the absolute value of difference in means between the given circadian parameter (e.g. period) for the two doses. The null distribution was generated by creating 10,000 random permutations of the dose labels and computing the test statistic for each permutation. The p-value was the fraction of statistics from the null distribution that were larger than the reference statistic.

To compare wound closure between doses, a one-tailed randomization test for longitudinal data was used [51]. It employs regression to test the null hypothesis that the dose did not affect the shape of the best-fit curve. For our data, the null hypothesis was more specifically that fitting a quadratic (with zero-intercept) to the data for both doses leads to a fit that is indistinguishable from fits from data for each individual dose. The reference test statistic was an F-statistic computed from the sum of the residuals for the pooled data in comparison to that for a fit to data from one dose. The null distribution was generated by un-correlating and shuffling the residuals and then computing the test statistic (see Storey et al, 2005 for more details; the process was repeated 1,000 times) [51]. The p-value was the fraction of the statistics from the null distribution that were smaller than the reference statistic. Multiple comparisons were corrected according to the Bonferroni method.

## Results

### Nobiletin affects circadian oscillations of *Per2*:*luc* and *Bmal1*:*luc* in U2OS, MCF7, and MDA-MB-231 cells in a cell-dependent manner

To assess the circadian effects of nobiletin in a detailed approach, we used luciferase-reporter cells for *Bmal1* and *Per2* promoter activity previously generated in our lab [37]. Here, we employed U2OS, MCF7, and MDA-MB-231 cell lines separately transfected with *Bmal1*:*luc* and *Per2*:*luc* [32,36]. Prior to initiating luminometry experiments, we evaluated potential viability effects of treatments to be used. The highest and lowest concentrations used in this study were determined based on multiple factors, including those used in previous studies, results from our viability assay (S1 Fig), and our desire to apply consistent nobiletin concentrations across cell lines. For reference, the concentration required for 50% induction of CYP1 enzyme activity in MCF7 cells is 44 μM [21]. IC_50_ values are not available for U2OS and MDA-MB-231 cells. Therefore, concentrations of 5 μM and 50 μM of nobiletin were used. While slightly diminished viability (~80%) was observed for U2OS cells treated at the highest concentration, none of the other cells or treatments resulted in significant changes (S1 Fig).

**Fig 1:**
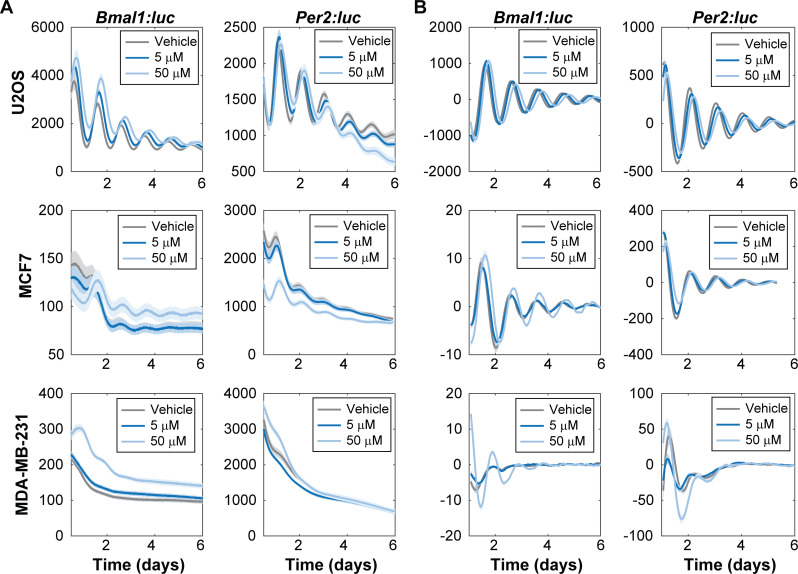
Nobiletin alters circadian dynamics in bone and breast cancer cell lines. Shown are **(A)** raw and **(B)** de-trended mean traces of (left) *Bmal1*:*luc* and (right) *Per2*:*luc* in (top) U2OS, (middle) MCF7, and (bottom) MDA-MB-231 cells, as obtained using luminometry. Each cell type was exposed to DMSO (vehicle), and 5 μM and 50 μM nobiletin conditions. The semi-transparent envelopes around the lines indicate the standard error of the mean (SEM). In several instances, the SEM is too small to extend beyond the line. **(B)** Each trace was de-trended by subtracting the mean of a 24-h sliding window and smoothed using the mean of a 3-h sliding window.

The same conditions were used to track circadian oscillations via reporter bioluminescence using real-time luminometry for 6 days (Fig 1 and S2–S5 Figs). A total of N = 12 replicates per condition per cell line/reporter was obtained, across three independent experiments, with N = 4 each. Consistent, rhythmic, anti-phase oscillations were observed for both *Bmal1*:*luc and Per2*:*luc* signals in U2OS and MCF7 (FFT-based test for rhythmicity [41], p < 0.05 for all *Per2*:*luc* recordings across all treatments, all *Bmal1*:*luc* recordings for treatments with 50 μM nobiletin, and 8 out of 12 *Bmal1*:*luc* recordings treated with DMSO and 5 μM nobiletin). However, in MDA-MB-231 cells, *Bmal1* and *Per2* reporters showed oscillations consistently only with 50 μM nobiletin treatment. These findings are consistent with previous results showing that MDA-MB-231 cells lack detectable rhythms while MCF7 are rhythmic [32]. The average time series from MDA-MB-231 cells (Fig 1A and S4C Fig) clearly shows elevated/enhanced raw bioluminescence for days 0–6 in the *Bmal1* reporter, and for approximately days 0–2 for the *Per2* reporter for 50 μM, but not for vehicle or the 5 μM treatment. De-trending reveals these deviations from the baseline and shows they are much larger for the 50 μM nobiletin condition versus the others; hence, the peak-to-trough amplitude is increased in MDA-MB-231 cells. We also confirmed that the treatment of cells with DMSO vehicle did not affect circadian oscillations, in comparison with non-treated cells (S6 and S7 Figs).

**Fig 2:**
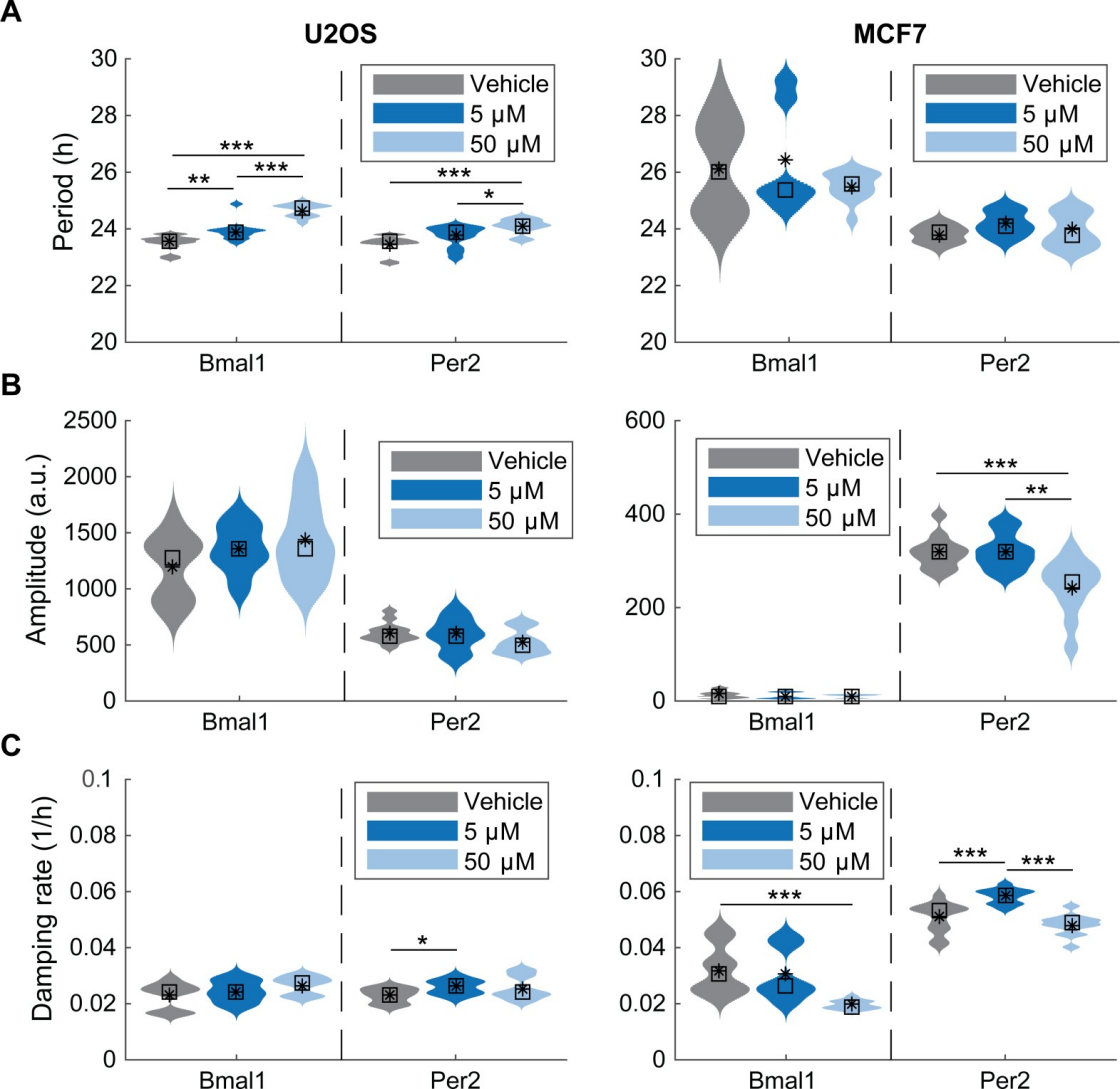
**Effects of nobiletin on circadian periods, amplitudes, and damping rates in (left) U2OS and (right) MCF7 cell lines.** Shown are the distributions of (**A**) period, (**B**) amplitude, and (**C**) damping rate parameters estimated from fitting a damped cosine curve to each de-trended bioluminescence recording. Within each subfigure, color indicates experimental condition, with results from *Bmal1*:*luc* shown on left and *Per2*:*luc* on the right (N = 12 per reporter per condition). The mean and median values are indicated with a black asterisk and square, respectively. Statistical significance was evaluated via a randomization test for difference in means with a Bonferroni correction (* p < 0.05, ** p < 0.01, *** p < 0.001).

We further evaluated the characteristics of data from U2OS and MCF7 cells (MDA-MB-231 cells were omitted due to lack of rhythmic patterns and are discussed further below). Our analyses show that nobiletin results in modestly increased circadian periods in both U2OS-*Bmal1*:*luc* and U2OS-*Per2*:*luc* cells (Fig 2), with higher concentrations yielding greater effects (randomization test for difference in means, Bonferroni-corrected p < 0.05). The changes to damping rates and amplitudes were not statistically significant, with the exception that 5 μM nobiletin treatment led to more damped *Per2*:*luc* oscillations. A repeated analysis with peak- and trough-finding methods for estimating the period, amplitude, and damping rate led to the same trends (S8–S11 Figs). For MCF7 cells, the trends in period, amplitude, or damping rate estimates across treatments varied, depending on the method used to estimate the properties (S8–S11 Figs).

### Nobiletin affects rhythmicity of MDA-MB-231 cell lines

**Fig 3:**
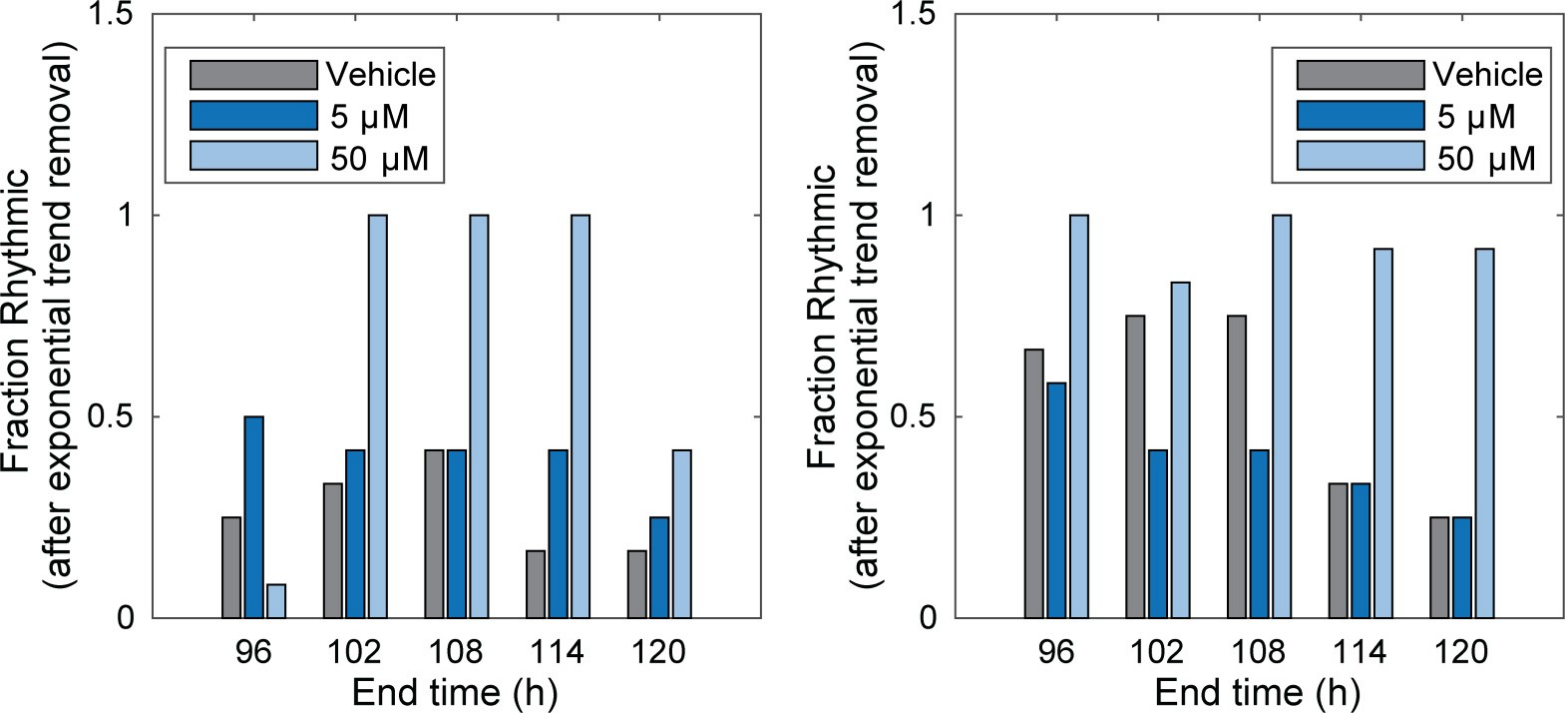
Nobiletin alters the rhythmicity of MDA-MB-231 cells. Shown are the fractions of recordings classified as rhythmic (p < 0.05 in FFT-based test), after removing an exponential trend, using time-series with increasing end times. The fraction scored rhythmic depends on how much of the time-series is included in the analysis, but cells treated with 50 μM nobiletin consistently scored as rhythmic more frequently than those treated with either DMSO or 5 μM. The only exception is *Per2*:*luc* recordings with the first 108 hours analyzed.

Previous work by our group has shown that even using real-time luminometry with *Bmal1* and *Per2* reporters, circadian oscillations remain undetectable (and arrhythmic) in MDA-MB-231 cells [32]. We find that this largely remains the case, however, at the highest concentration of nobiletin treatment (50 μM), rhythmicity was found to be enhanced in both *Bmal1*:*luc* and *Per2*:*luc* cells. This was determined by applying an FFT-base test for rhythmicity [50] to recordings after removing an exponential (Fig 3) or quadratic (S12 Fig) trend. The results varied depending on the length of time-series considered, but recordings from cells treated with 50 μM nobiletin consistently scored rhythmic at a higher rate than all others. Visual inspection of the de-trended time-series confirms that nobiletin enhances rhythmicity (S13–S16 Figs). To assess whether nobiletin resulted in enhanced overall transcription of *Bmal1* or *Per2*, we conducted an RT-PCR experiment with MDA-MB-231, U2OS, and MCF7 cells (S17 Fig). We found that no significant average changes occurred following nobiletin treatment across all of the cell lines, which were not synchronized.

### Effects of nobiletin on oncogenic characteristics vary by cell type

**Fig 4:**
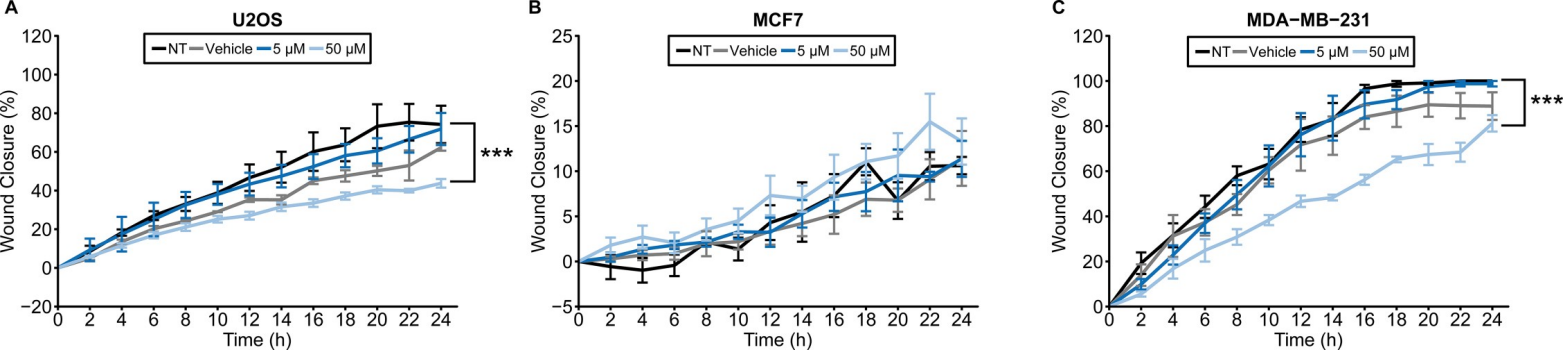
Effects of nobiletin on cellular migration of bone and breast cancer cells. A wound healing/scratch assay was performed with (**A**) U2OS, (**B**) MCF7, and (**C**) MDA-MB-231 cells. Nobiletin reduced cell migration in U2OS and MDA-MB-231 cell lines at 50 μM concentration. No substantial changes were observed in MCF7 cells. Error bars represent SEM. Statistical significance was evaluated via comparison of longitudinal data (*** p < 0.001). NT = non-treated; Vehicle = DMSO-only control (0.2%).

Previous studies showed that nobiletin inhibits the proliferation of U2OS, MCF7, and MDA-MB-231 cells [21,23,26] and reduces their migration and invasion [22,23,26,30]. As nobiletin has been posited to have therapeutic potential, we were interested in determining whether its effects were similar or differed by cell line. First, we assessed changes to cellular migration following nobiletin treatment via wound-healing/scratch assay (Fig 4). The same concentrations were used as in our assessments of circadian rhythms. In U2OS and MDA-MB-231 cells, migration decreased in samples treated with 50 μM nobiletin (p < 0.001 comparing longitudinal data with replicates, as in Storey et al, 2005) [51]. In MDA-MB-231 cells, there was a 29.5% relative difference in mean wound closure between t = 12 h and t = 22 h for nobiletin compared to vehicle-treated samples; the difference between samples was slighter at the conclusion of the experiment (t = 24 h). In contrast, the effect on U2OS cells was more subtle, with a 20.9% difference in mean wound closure between t = 12 h and t = 22 h, although the effect was sustained through the final time-point. Nobiletin had no significant effect on migration of MCF7 cells.

**Fig 5:**
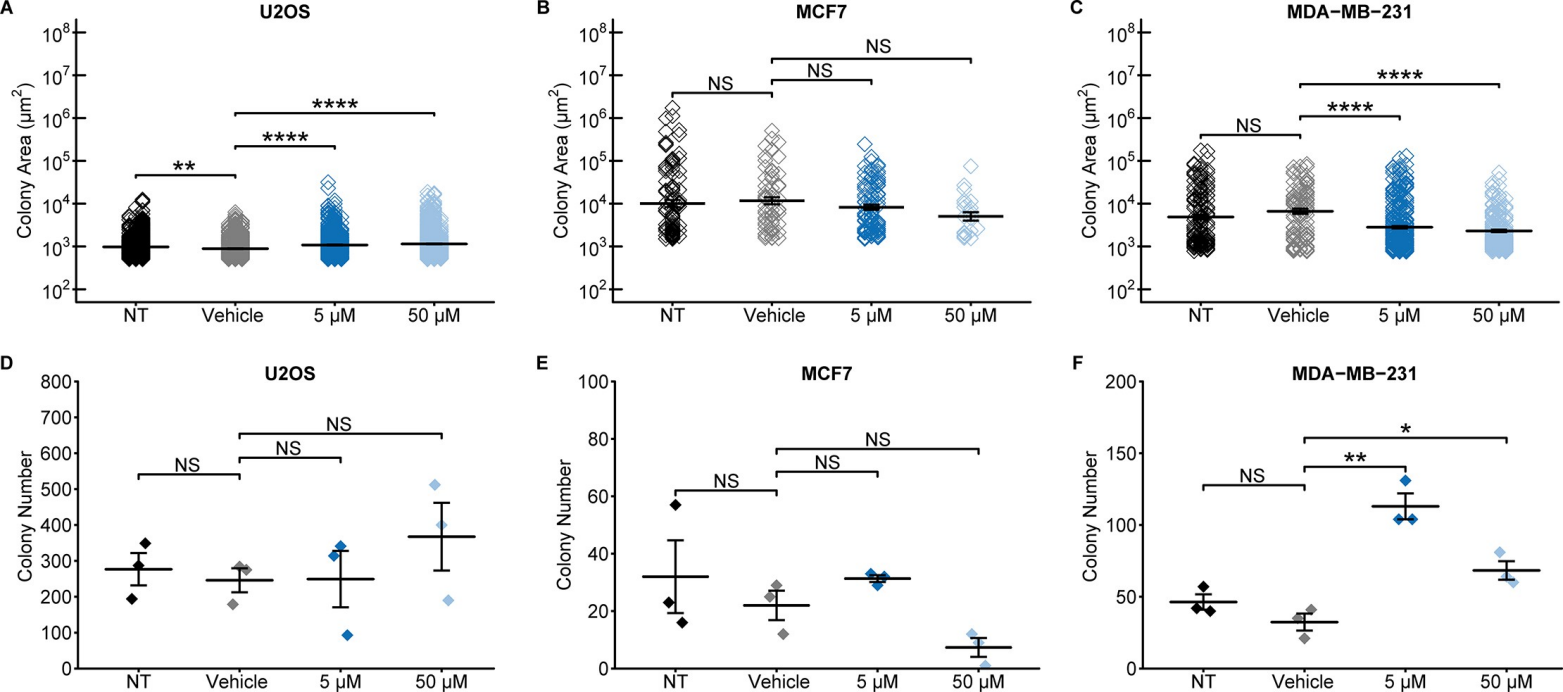
Nobiletin alters colony area and number for bone and breast cancer cells. Following colony formation assays, colony areas were evaluated for (**A**) U2OS, (**B**) MCF7, and (**C**) MDA-MB-231 cells. Nobiletin increased colony areas of U2OS cells in a statistically significant manner, did not affect colony area in MCF7 cells, and most substantially reduced colony areas in a dose-dependent manner in MDA-MB-231 cells. Colony numbers were not affected in (**D**) U2OS or (**E**) MCF7 cells, and significantly altered by both treatments in (**F**) MDA-MB-231 cells. Error bars represent SEM. Statistical significance was evaluated via Student’s t-test with a Bonferroni correction (* p < 0.05, ** p < 0.01, *** p < 0.001, **** p < 0.0001). NT = non-treated; Vehicle = DMSO-only control (0.2%), NS = not significant.

We used a colony formation assay to determine whether nobiletin could affect anchorage-independent colony formation in a three-dimensional environment. In this experiment, we evaluated both colony area and size. In U2OS cells, colony areas surprisingly increased relative to the vehicle-treated controls, by 36% and 50% for 5 and 50 μM treatments, respectively (Fig 5A and S18 Fig). However, the numbers of colonies present did not change compared to the vehicle-treated samples (Fig 5D). In MCF7 cells, nobiletin did not significantly alter the area or number of colonies (Fig 5B and 5E and S18 Fig). The most substantial changes in colony size were observed in MDA-MB-231 cells, whose areas decreased by 49% and 73% for 5 and 50 μM treatments, respectively, relative to the vehicle control (Fig 5C and S18 Fig). In both cases, we also found increased colony numbers (Fig 5F). Decreased colony sizes may be accompanied by increased numbers because it is possible that nobiletin prevents the aggregation of smaller colonies into larger ones, which can result in higher numbers of smaller colonies but fewer larger ones [36].

## Discussion

Nobiletin is a small molecule with a range of therapeutic effects whose influence on circadian rhythms has recently been uncovered. While numerous studies have evaluated its efficacy in various disease models, to our knowledge only one has assessed therapeutic and circadian effects concomitantly, addressing metabolic syndrome [2]. Because nobiletin has been shown to result in beneficial outcomes in multiple cancer types, we considered it important to study whether a correlation may exist between its anti-oncogenic and circadian changes. Circadian rhythm disruption has been found to be associated with cancers and their aggressiveness. As examples, peripheral blood mononuclear cells (PBMCs) from healthy individuals had circadian oscillations while chronic myeloid leukemia did not [52], low tumor grade MCF7 cells had circadian rhythms while high tumor grade MDA-MB-231 cells were arrhythmic [32], and in models of disease progression, circadian discrepancies were observed in both breast [37] and colon carcinoma cells [53].

In this study, we assessed how nobiletin affects circadian and cancerous characteristics in three different cell lines. We hypothesized that nobiletin’s effects on oncogenic features, including motility and anchorage-independent colony formation, would be accompanied by changes in circadian rhythms, and that their extents may be linked. We employed U2OS, MCF7, and MDA-MB-231 cells; while the first two possess strong and weaker oscillations, respectively, MDA-MB-231 cells had been previously found to be arrhythmic [32]. The application of luciferase reporters to track circadian rhythms has been widely used [2,3,28,31,32,37,54,55] on account of their ability to elucidate even subtle circadian oscillations in a higher resolution manner [32,37,54]. Raw data is typically detrended to remove artificial trends or baselines associated with readings; various methods have been applied, and this is a generally accepted part of circadian rhythm analysis [55–58]. It also assists in visualization of subtle oscillations that cannot be observed in raw data due to the large scale of signal damping compared to the magnitude of the oscillation being observed [32,55–58]. Following treatment with nobiletin, U2OS and MCF7 showed subtle circadian alterations and slight changes to cellular characteristics. In contrast, the MDA-MB-231 responses included a clear enhancement of circadian oscillations and significant reduction in cell migration and colony area.

Previous studies using clock knockout and normal MEF cells showed amplitude enhancements upon nobiletin treatment [2,3]. These effects are hypothesized to be a result of the activation by nobiletin of RORα and RORγ, transcriptional activators of the *Bmal1* gene. In our work, U2OS-*Bmal1*:*luc*, and MDA-MB-231 *Bmal1*:*luc* and *Per2*:*luc* cell lines showed amplitude enhancements, with rescue of rhythmicity in MDA-MB-231 cells. However, no amplitude changes were observed in U2OS-*Per2*:*luc* cells and nobiletin subtly reduced circadian amplitudes of both MCF7 *Bmal1*:*luc* and *Per2*:*luc* cell lines. Our raw data supports these findings, with the exception of MCF7 *Bmal1*:*luc*, where bioluminescence was enhanced. Nobiletin’s enhancement of circadian amplitude may vary depending on initial characteristics. For example, significant amplitude enhancement was observed previously in MEFs where CLOCK was knocked-out [2], while standard MEFs showed more minor effects [3]. It is possible that the outcomes of nobiletin treatment are dependent on circadian (dys)function. We observed that rhythmic U2OS and MCF7 cells showed subtle changes, while arrhythmic MDA-MB-231 cells showed clear alterations with nobiletin. Those cell lines where the clock was clearly functioning benefitted less than the one where it was not.

In terms of period, prior work showed that nobiletin increases the period of *PER2*::*Luc* MEFs [2,3], and mouse liver slices [3]. We observed similar but subtle effects in both *Bmal1* and *Per2* U2OS reporter cells. However, MCF7 cells had no significant changes in period. In MDA-MB-231 cells, periods could not be determined for arrhythmic oscillations, but for rhythmic oscillations periods were shown to be within the circadian range. Our data showed different circadian effects between the two reporters associated with the same cell line to same nobiletin treatments (e.g. the periods of U2OS-*Bmal1*:*luc* versus -*Per2*:*luc* and the periods and amplitudes of MCF7-*Bmal1*:*luc* versus *-Per2*:*luc* cell lines). Such disparate responses from reporters in the same cell type have been seen previously in U2OS and NIH 3T3 cell lines [59]. This could be a result of having each reporter in a separate cell line. Therefore, in the future, tracking both *Bmal1* and *Per2* rhythms concomitantly using a dual-luciferase reporter system will be helpful.

Nobiletin also affected oncogenic features of the cancer cells to varying extents. Sheet migration is a process where cancer cells migrate in two dimensions during metastasis, and can be assessed via wound healing assay [60]. Furthermore, normal cells rely on contacts between cells and extra cellular matrix for cell growth and division, while more aggressive ones can grow without it. The ability of this anchorage-independent cell proliferation can be measured using a colony formation assay [61]. The colony counts and areas obtained from this assay are quantitative values that can determine tumorigenicity of a cell line [62]. In this study, we measured the ability of cancer cells to migrate and proliferate in an anchorage-independent manner in three dimensions [61] using both of these methods. We saw significantly reduced cell migration at the highest concentration of nobiletin treatment in U2OS and MDA-MB-231 cells, but not in MCF7 cells.

Many of the pathways associated with nobiletin’s anti-migration and–proliferation activities in the three cell lines tested are connected to the circadian clock, or can be connected to its ROR target. In prior evaluation of nobiletin’s circadian effects, the presence of functional RORα and RORγ was required [2]. A previous study showed that nobiletin reduced cell migration (to a similar extent as herein) via downregulation of matrix metallopeptidases (MMPs) and nuclear factor kappa B (NF-κB) protein levels in U2OS cells [26]. MMPs are repressed by the circadian protein PER [63], while NF-κB has been shown to be downregulated by ROR [64]. Hence, ROR activation and enhanced clock activity may affect migration. Our data shows that nobiletin also reduced MDA-MB-231 migration at the highest concentration tested; a previous study (using a higher concentration, 200 μM) showed that nobiletin inhibits motility by affecting cluster of differentiation 36 (CD36), phospho-signal transducer and activator of transcription 3 (pSTAT3), and NF-κB proteins [30]. STAT3 has shown circadian rhythms in rat suprachiasmatic nuclei (SCN) [65,66], and it is possible that nobiletin affects its oscillations as well. In our study, nobiletin did not alter MCF7 cell migration at the highest concentration tested (50 μM). However, previous studies showed that it reduced cell migration at concentrations of 50 μM [22], 100 μM and 200 μM [23]. Multiple experiments showed that nobiletin down-regulates CD36, STAT3, pSTAT3, MMPs, and NF-κB proteins, among others in MCF7 cells [22,23,30].

Colony areas concomitantly increased and decreased with dose in U2OS and MDA-MB-231 cells, respectively, while MCF7 was not affected. Previous studies showed that nobiletin-treated breast cancer cells had reduced cell proliferation in largely two-dimensional assays [21,24]. In one, nobiletin caused G1 cell cycle block in MCF7 cells at 75 μM and 100 μM concentrations [21], while in another it arrested MCF7 cells in the G0/G1 phases of the cell cycle at 50 μM and decreased ERK activation and suppressed cyclin D1 expression [67]. Cyclin D1 is a key regulator of G_0_/G_1_ cell-cycle checkpoint, and is under circadian regulation by a PER1 protein complex [68]. Hence, circadian enhancement via nobiletin may be involved. Another study using MCF7 and MDA-MB-231 cells also showed that nobiletin reduced cell proliferation in the concentration range of 50 μM-300 μM, and affected the proto-oncogene tyrosine-protein kinase (SRC)/focal adhesion kinase (FAK)/STAT3 pathway [23]. To the best of our knowledge, nobiletin has been evaluated only in terms of three-dimensional growth in MCF7 cells using a sphere formation assay, where significant reductions in size were observed at 200 μM [30]. We also found decreased colony areas at the highest concentration tested. Colony formation assays in other cancer cell lines such as ACHN and Caki2, showed that nobiletin dose-dependently reduced colony numbers at concentrations up to 120 μM [18], and colony numbers of H460 non-small cell lung cancer cells at 50 μM [69].

Interestingly, in our study, we found that nobiletin yields different circadian and anti-cancer effects (specifically reduction of cell migration in two-dimensions and formation of anchorage independent colonies), depending on cell line. Another significant difference among these cells is the levels of nobiletin metabolizing protein, Cytochrome P450 (CYP), present. CYP metabolizes naturally occurring flavonoids; the metabolized products often have higher activities compared to parent compounds [21,69]. Nobiletin has been shown to be metabolized via CYP1 [21,24]. While CYP1 protein levels in U2OS cells have not yet been determined, MDA-MB-231s have higher CYP1 expression compared to MCF7 [70]. While the IC_50_ of nobiletin has not been determined in MDA-MB-231 cells, in another triple negative cell line where CYP1 was shown to be constitutively expressed, MDA-MB-468 [24], the IC_50_ of nobiletin is 0.1 ± 0.04 μM [24]. In contrast, in MCF7 cells it is 44 μM [21]. In the future, it would be useful to determine whether nobiletin’s metabolites are responsible for its activity, and to directly compare its effects in these cell lines in parallel.

In our study, we found that nobiletin can both induce circadian rhythmicity and reduce the oncogenic features of cell migration in a two-dimensional layer and formation of anchorage independent colonies in MDA-MB-231 cells. While our work focused on *in vitro* assessments, others have observed that inductions of rhythms via different means can also affect tumors in *in vivo* models. One study used a cyclin-dependent kinase inhibiting small molecule, Seliciclib, that induced rhythmic clock gene expression and reduced tumor growth in Glasgow osteosarcoma tumors in mice [71]. Two others used established methods for inducing/synchronizing rhythms, and showed that they too could affect tumors *in vivo*. Kiessling et al showed that induction of circadian rhythmicity via dexamethasone, forskolin or heat shock all slowed B16 and HCT-116 tumor growth *in vivo*, [45] while Li *e*t al demonstrated that induction of rhythms based on meal time reduced P03 pancreatic adenocarcinoma tumor weight in mice [72].

We conclude that alteration to cancer cell circadian rhythms and oncogenic features via nobiletin treatment depend on cell type. We have observed that cells with greater circadian changes also have more substantial effects on cellular characteristics, and vice-versa. In the future, it is highly merited to study whether there is a direct relationship between circadian alterations (rescue/enhancement) and affecting oncogenic features in cancer cells. Furthermore, because the densities of cell populations used can also affect the extent(s) to which effects are observed due to growth stage, cell-cell interactions, and metabolic status [73,74], nobiletin should also be evaluated in cells with differing levels of confluence.

## Supporting information

S1 Fig Cell viability assay. **(A)** U2OS, **(B)** MDA-MB-231, and **(C)** MCF7 cells were treated under conditions shown. Viability was determined using Alamar blue assay. No significant differences were observed in nobiletin-treated samples compared to vehicle (0.2% DMSO) samples, with the exception of the 50 μM U2OS cells. Error bars represent standard deviations across three biological replicates. Statistical significance was evaluated via two tailed Student’s t-test (** Bonferroni-corrected p<0.01). NT = non-treated.
(TIF)

S2 Fig Effects of nobiletin on circadian dynamics in bone and breast cancer cell lines. Replicates of the luminometry data shown in **Fig 1** are presented here, for (left) *Bmal1*:*luc* and (right) *Per2*:*luc* in (**A**) U2OS, (**B**) MCF7, and (**C**) MDA-MB-231 cells. Each cell line was treated with vehicle (0.2% DMSO), and 5 μM and 50 μM nobiletin conditions. N = 12 replicates were obtained for each treatment and cell line. Data were evaluated beginning from t = 0.5 (day); each trace has been de-trended by subtracting the mean of a 24h sliding window and smoothed using the mean of a 3-h sliding window, making the de-trended date begin at t = 1 (days).
(TIF)

S3 Fig Effects of nobiletin on circadian dynamics in bone and breast cancer cell lines. We show the individual replicates depicted in **S2 Fig**, further separated by condition and date. N = 4 replicates were obtained for each treatment and cell line, on each of three separate dates (indicated in the legends). Data were evaluated beginning from t = 0.5 (day); each trace has been de-trended by subtracting the mean of a 24h sliding window and smoothed using the mean of a 3-h sliding window, making the de-trended date begin at t = 1 (days).
(TIF)

S4 Fig Effects of nobiletin on circadian dynamics in bone and breast cancer cell lines. Shown are the raw data for (left) *Bmal1*:*luc* and (right) *Per2*:*luc* traces in (**A**) U2OS, (**B**) MCF7, and (**C**) MDA-MB-231 cells under vehicle (0.2% DMSO), and 5 μM and 50 μM nobiletin treatment conditions. N = 12 replicates were obtained for each treatment and cell line.
(TIF)

S5 Fig Effects of nobiletin on circadian dynamics in bone and breast cancer cell lines. We show the individual replicates of raw data depicted in **S4 Fig**, further separated by condition and date. N = 4 replicates were obtained for each treatment and cell line, on each of three separate dates (indicated in the legends).
(TIF)

S6 Fig Vehicle treatment has no effect on circadian oscillations. Shown are the smoothed and de-trended replicates for *Bmal1*:*luc* and *Per2*:*luc* reporters in (**A**) U2OS, (**B**) MCF7, and (**C**) MDA-MB-231 cells under non-treated (black) and vehicle-treated (gray) conditions. N = 4 for each treatment for each cell line. Data were evaluated beginning from t = 0.5 (day); each trace has been de-trended by subtracting the mean of a 24 h sliding window and smoothed using the mean of a 3 h sliding window, making the de-trended date begin at t = 1 (days).
(TIF)

S7 Fig Vehicle treatment has no effect on circadian oscillations. Shown are raw data replicates for *Bmal1*:*luc* and *Per2*:*luc* reporters in (**A**) U2OS, (**B**) MCF7, and (**C**) MDA-MB-231 cells under non-treated (black) and vehicle-treated (gray) conditions. N = 4 for each treatment for each cell line.
(TIF)

S8 Fig Multiple period-estimation methods reveal similar trends for U2OS *Bmal1*:*luc* and *Per2*:*luc* and MCF7 *Per2*:*luc* recordings, but not MCF7 *Bmal1*:*luc*. Shown are the distributions of period estimates for each recording (N = 12 for each condition for each reporter and cell line). For each cell line (U2OS on left, MCF7 on right) and each reporter (*Bmal1* on left, *Per2* on right), we show the period distribution, color-coded by treatment (gray = vehicle, blue = 5 μM nobiletin, light blue = 50 μM nobiletin). Each row shows results from a different method (DC = damped cosine-fitting; the remaining method names indicate the phase marker used to estimate the period, e.g. “peak” indicates the difference in time between the peak of each cycle is used). Randomization tests for difference in means were performed to determine if treatment led to statistically significant differences. P-values were corrected according to Bonferroni’s method (* p < 0.05, ** p < 0.01, *** p < 0.001). For U2OS (both reporters) and MCF7 *Per2*:*luc*, the period estimates of 50 μM nobiletin treated recordings are either longer than or not statistically significantly different from those treated with vehicle or 5 μM. For MCF7 *Bmal1*:*luc*, however, two methods (trough-to-trough estimates and mean-crossing while levels are rising) indicate that the 50 μM nobiletin treatment leads to shorter periods and one (mean-crossing while levels are falling) indicates that it leads to longer periods.
(TIF)

S9 Fig Multiple amplitude-estimation methods reveal few trends for *Bmal1*:*luc*, the same trends for U2OS *Per2*:*luc*, and conflicting trends for MCF7 *Per2*:*luc*. Shown are the distributions of amplitude estimates for each recording (N = 12 for each condition for each cell line). For each cell line (U2OS on left, MCF7 on right) and each reporter (*Bmal1* on left, *Per2* on right), we show the amplitude distribution, color-coded by treatment (gray = vehicle, blue = 5 μM nobiletin, light blue = 50 μM nobiletin). Each row shows results from a different method (DC = damped cosine-fitting; Cycle1 = difference between magnitudes of first peak and first trough; Cycle2 = difference between magnitudes of second peak and second trough). Randomization tests for difference in means were performed to determine if treatment led to statistically significant differences. P-values were corrected according to Bonferroni’s method (* p < 0.05, ** p < 0.01, *** p < 0.001). For U2OS *Bmal1*:*luc*, there are no trends. For U2OS *Per2*:*luc*, 50 μM treatment led to a smaller amplitude. For MCF7 *Bmal1*:*luc*, only the amplitude of the second cycle is different across treatments, with a 50 μM treatment leading to a higher amplitude. For MCF7 *Per2*:*luc*, 50 μM treatment led to a smaller amplitude.
(TIF)

S10 Fig Multiple damping-estimation methods reveal moderate trends for both U2OS *Bmal1*:*luc* and for MCF7. Shown are the distributions of damping estimates for each recording (N = 12 for each condition for each cell line). For each cell line (U2OS on left, MCF7 on right) and each reporter (*Bmal1* on left, *Per2* on right), we show the amplitude distribution, color-coded by treatment (gray = vehicle, blue = 5 μM nobiletin, light blue = 50 μM nobiletin). Each row shows results from a different method (DC Damping rate = damped cosine-fitting; Amplitude Loss = 1 –Cycle2 Amplitude/Cycle1 Amplitude). Randomization tests for difference in means were performed to determine if treatment led to statistically significant differences. P-values were corrected according to Bonferroni’s method (* p < 0.05, ** p < 0.01, *** p < 0.001). For U2OS *Bmal1*:*luc* and *Per2*:*luc*, 50 μM nobiletin treatment leads to increased amplitude loss. For MCF7, 50 μM nobiletin treatment reduces damping (as determined both by cosine-fitting and by measuring amplitude loss from cycle 1 to cycle 2).
(TIF)

S11 Fig Agreement of period-estimation methods is consistent for U2OS and mildly inconsistent for MCF7. For a given reporter and treatment, each of 12 replicates had its period estimated by 5 methods (damped-sine fitting, peak-to-peak, trough-to-trough, mean-crossing-to-mean-crossing on the rise, mean-crossing-to-mean-crossing on the fall). The coefficient of variation (CV) across the 5 methods is computed. Shown are the distributions of CVs for (**A**) U2OS and (**B**) MCF7 *Bmal1*:*luc* and *Per2*:*luc* recordings. Color indicates treatment (gray = vehicle, blue = 5 μM nobiletin, light blue = 50 μM nobiletin). For U2OS, the CV of period estimation is less than 4%. For MCF7 *Bmal1*:*luc*, vehicle treatment and 5 μM nobiletin period estimates vary more (the standard deviation is 10% of the mean).
(TIF)

S12 Fig Nobiletin alters the rhythmicity of MDA-MB-231 cells. Shown are the fractions of recordings classified as rhythmic (p < 0.05 in FFT-based test), after removing a quadratic trend, using time-series with increasing end times. The fraction scoring as rhythmic depends on the extent of the time-series included in the analysis, but cells treated with 50 μM nobiletin consistently scored as rhythmic more frequently than those treated with either vehicle or 5 μM. The only exception is for *Bmal1*:*luc* recordings with the first 96 hours analyzed.
(TIF)

S13 Fig Removing a quadratic trend from the first four days shows that nobiletin alters the rhythmicity of MDA-MB-231 cells. **(A)** Shown are luminometry recordings after removing a quadratic trend from the first 96 hours of data. Subfigure placement and color indicate treatment (top/gray = vehicle, middle/blue = 5 μM nobiletin, bottom/light blue = 50 μM nobiletin). **(B)** Shown are the fractions of recordings classified as rhythmic by an FFT-based test, using increasing p-value cut-offs. For *Bmal1*:*luc*, both vehicle-treated and 50 μM nobiletin-treated cells are scored as rhythmic, but visual inspection demonstrates that the rhythm is most prominent for recordings from the 50 μM nobiletin treatment. For *Per2*:*luc*, visual inspection and the fraction scored as rhythmic both indicate that rhythms are present in cells treated with 50 μM nobiletin.
(TIF)

S14 Fig Removing a quadratic trend from the first five days shows that nobiletin alters the rhythmicity of MDA-MB-231 cells. **(A)** Shown are luminometry recordings after removing a quadratic trend from the first 120 hours of data. Subfigure placement and color indicate treatment (top/gray = vehicle, middle/blue = 5 μM nobiletin, bottom/light blue = 50 μM nobiletin). **(B)** Shown are fractions of recordings classified as rhythmic by an FFT-based test, using increasing p-value cut-offs. Visual inspection and the fraction scored as rhythmic (when p = 0.05) both indicate that rhythms are present in cells treated with 50 μM nobiletin. For *Per2*:*luc*, this pattern is consistent, regardless of the size of the p-value. For *Bmal1*:*luc*, both vehicle-treated and 50 μM nobiletin-treated cells scored as rhythmic, but visual inspection demonstrates that the rhythm is most prominent for recordings from the 50 μM nobiletin treatment. For *Per2*:*luc*, visual inspection and the fraction scored as rhythmic both indicate that rhythms are present in cells treated with 50 μM nobiletin.
(TIF)

S15 Fig Removing an exponential trend from the first four days shows that nobiletin alters the rhythmicity of MDA-MB-231 cells. **(A)** Shown are luminometry recordings after removing an exponential trend from the first 96 hours of data. Subfigure placement and color indicate treatment (top/gray = vehicle, middle/blue = 5 μM nobiletin, bottom/light blue = 50 μM nobiletin). **(B)** Shown are fractions of recordings classified as rhythmic by an FFT-base test, using increasing p-value cut-offs. For *Bmal1*:*luc*, both vehicle- and 50 μM nobiletin-treated cells scored as rhythmic, but visual inspection demonstrates that the rhythm is most prominent for recordings from the 50 μM nobiletin treatment. For *Per2*:*luc*, visual inspection and the fraction scored as rhythmic both indicate that rhythms are present in cells treated with 50 μM nobiletin.
(TIF)

S16 Fig Removing an exponential trend from the first five days shows that nobiletin alters the rhythmicity of MDA-MB-231 cells. **(A)** Shown are luminometry recordings after removing an exponential trend from the first 120 hours of data. Subfigure placement and color indicate treatment (top/gray = vehicle, middle/blue = 5 μM nobiletin, bottom/light blue = 50 μM nobiletin). **(B)** Shown are the fractions of recordings classified as rhythmic by and FFT-base test, using increasing p-value cut-offs. Visual inspection and the fraction scored as rhythmic (when p = 0.05) both indicate that rhythms are present in cells treated with 50 μM nobiletin. For *Per2*:*luc*, this pattern is consistent, regardless of the size of the p-value.
(TIF)

S17 Fig Nobiletin does not increase *Bmal1* or *Per2* mRNA levels in U2OS, MCF7, or MDA-MB-231 cells. mRNA levels were quantified using RT-PCR. No significant differences were observed in nobiletin treated samples compared to vehicle treated samples in any of the cell lines tested. Each treatment contained three biological replicates, with three technical replicates each. Error bars represent SEM. Statistical significance was evaluated via two tailed t-test in R ggpubr library under equal variance, and 0.95 confidence interval (* p < 0.05). NT = non-treated; Vehicle = 0.2% DMSO.
(TIF)

S18 Fig Representative images from colony formation assay. A single image for each treatment and cell line is shown. Scale bars are 106 μm.
(TIF)
