# Supplementary figures and images for: Nobiletin affects circadian rhythms and oncogenic characteristics in a cell-dependent manner
Source: PLoS One. 2020 Jul 24;15(7):e0236315. doi: 10.1371/journal.pone.0236315 (PMC7380617; doi:10.1371/journal.pone.0236315)

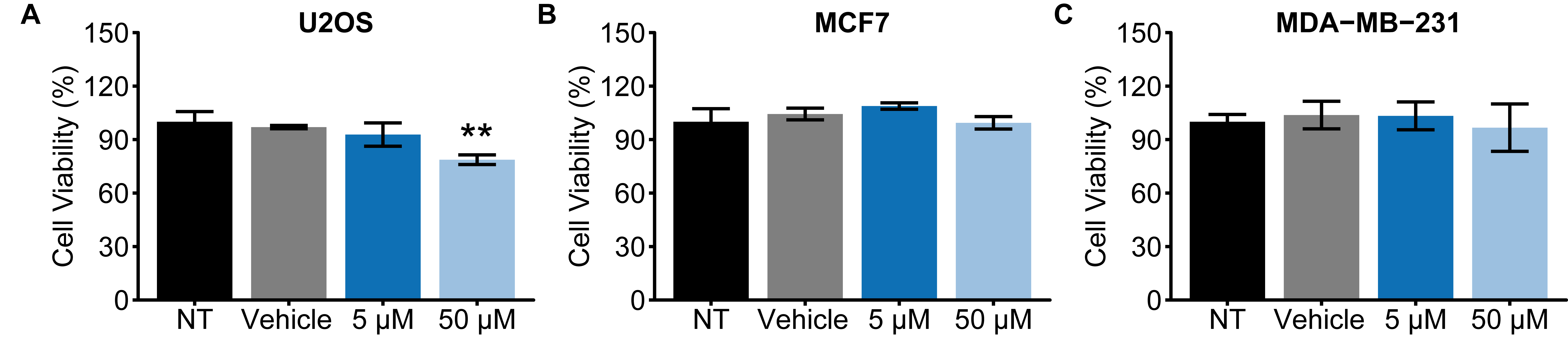

Supplement: S1 Fig — (A) U2OS, (B) MDA-MB-231, and (C) MCF7 cells were treated under conditions shown. Viability was determined using Alamar blue assay. No significant differences were observed in nobiletin-treated samples compared to vehicle (0.2% DMSO) samples, with the exception of the 50 μM U2OS cells. Error bars represent standard deviations across three biological replicates. Statistical significance was evaluated via two tailed Student’s t-test (** Bonferroni-corrected p<0.01). NT = non-treated. (TIF) [file pone.0236315.s001.tif]

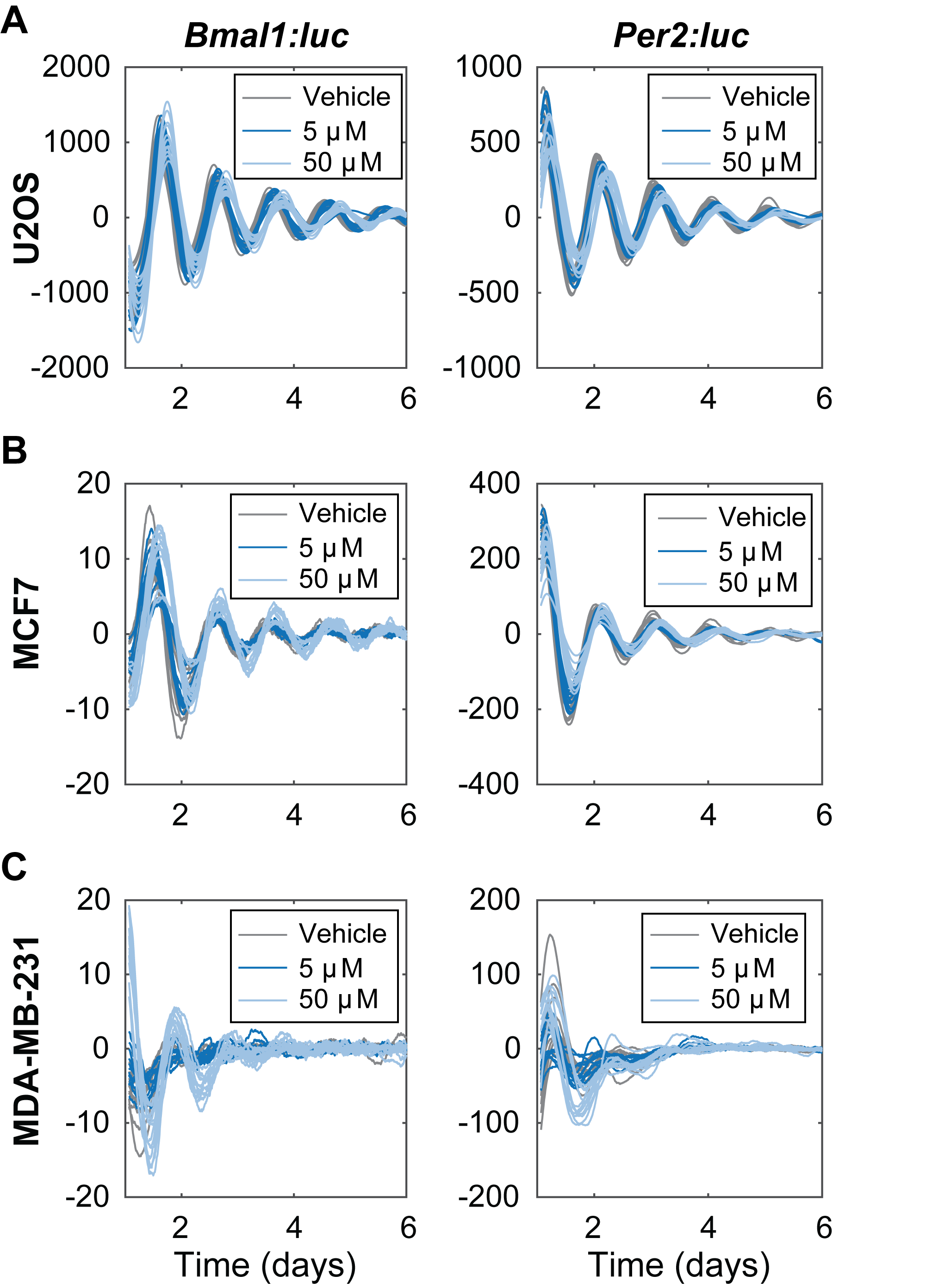

Supplement: S2 Fig — Replicates of the luminometry data shown in Fig 1 are presented here, for (left) Bmal1:luc and (right) Per2:luc in (A) U2OS, (B) MCF7, and (C) MDA-MB-231 cells. Each cell line was treated with vehicle (0.2% DMSO), and 5 μM and 50 μM nobiletin conditions. N = 12 replicates were obtained for each treatment and cell line. Data were evaluated beginning from t = 0.5 (day); each trace has been de-trended by subtracting the mean of a 24h sliding window and smoothed using the mean of a 3-h sliding window, making the de-trended date begin at t = 1 (days). (TIF) [file pone.0236315.s002.tif]

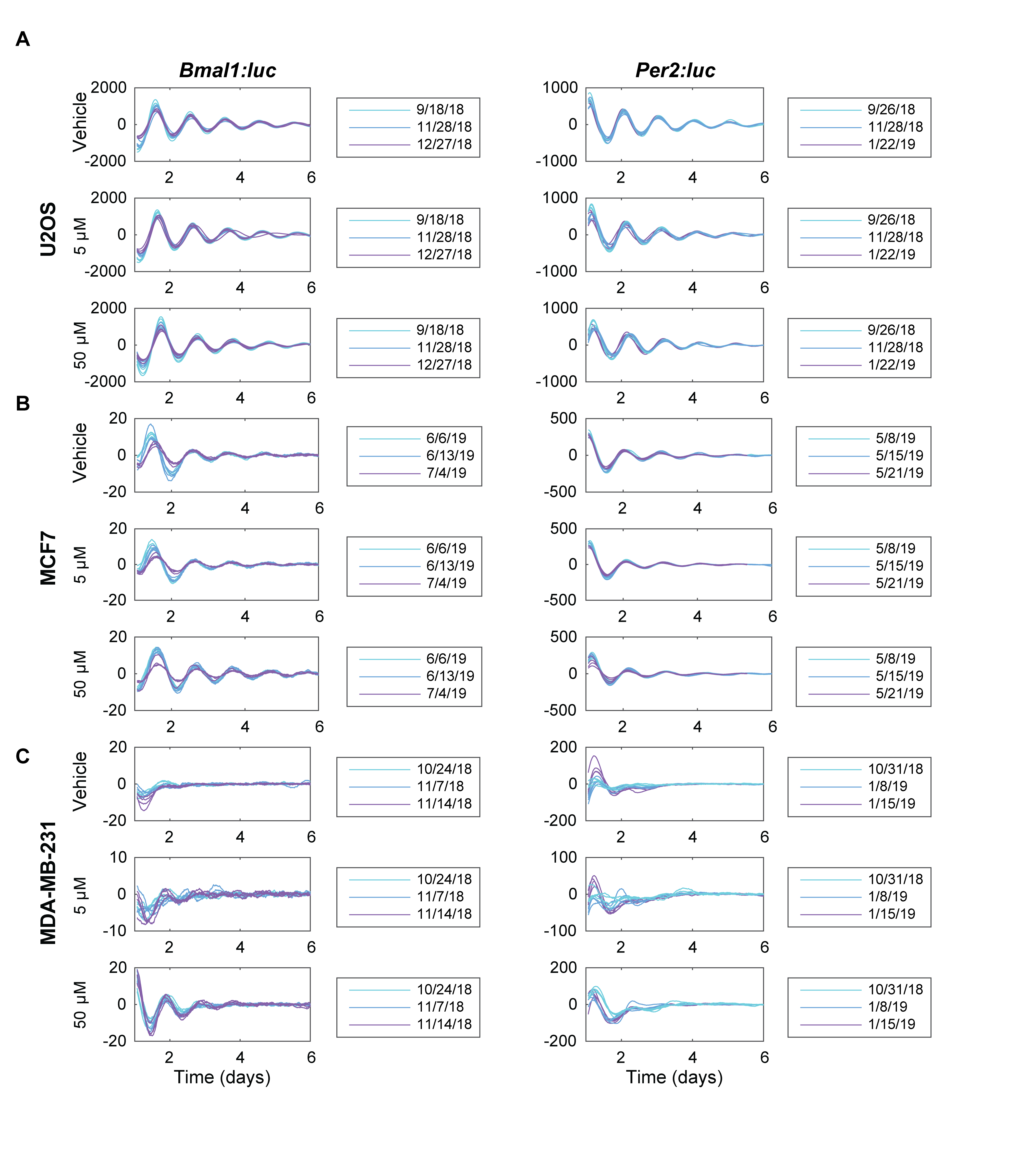

Supplement: S3 Fig — We show the individual replicates depicted in S2 Fig, further separated by condition and date. N = 4 replicates were obtained for each treatment and cell line, on each of three separate dates (indicated in the legends). Data were evaluated beginning from t = 0.5 (day); each trace has been de-trended by subtracting the mean of a 24h sliding window and smoothed using the mean of a 3-h sliding window, making the de-trended date begin at t = 1 (days). (TIF) [file pone.0236315.s003.tif]

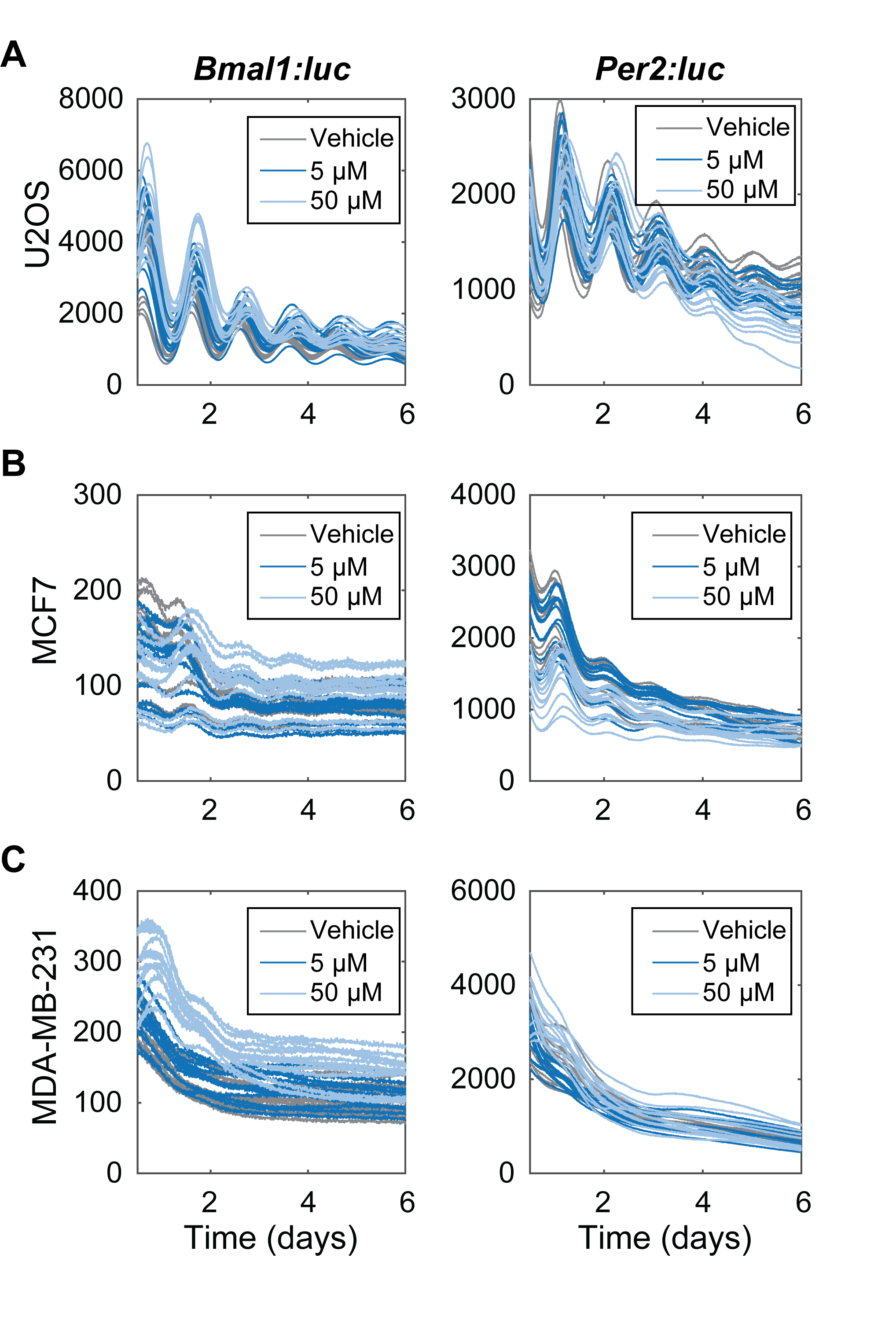

Supplement: S4 Fig — Shown are the raw data for (left) Bmal1:luc and (right) Per2:luc traces in (A) U2OS, (B) MCF7, and (C) MDA-MB-231 cells under vehicle (0.2% DMSO), and 5 μM and 50 μM nobiletin treatment conditions. N = 12 replicates were obtained for each treatment and cell line. (TIF) [file pone.0236315.s004.tif]

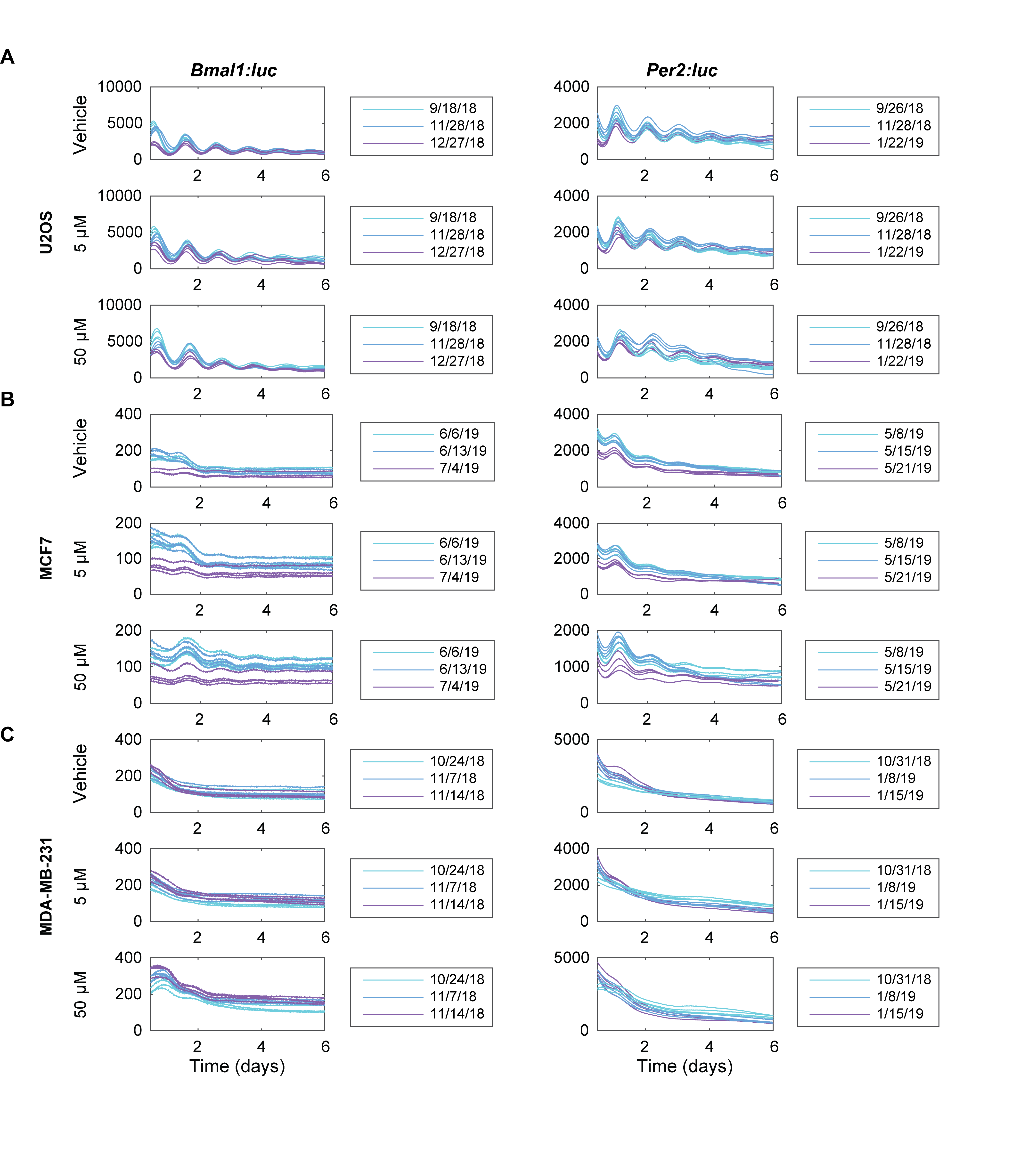

Supplement: S5 Fig — We show the individual replicates of raw data depicted in S4 Fig, further separated by condition and date. N = 4 replicates were obtained for each treatment and cell line, on each of three separate dates (indicated in the legends). (TIF) [file pone.0236315.s005.tif]

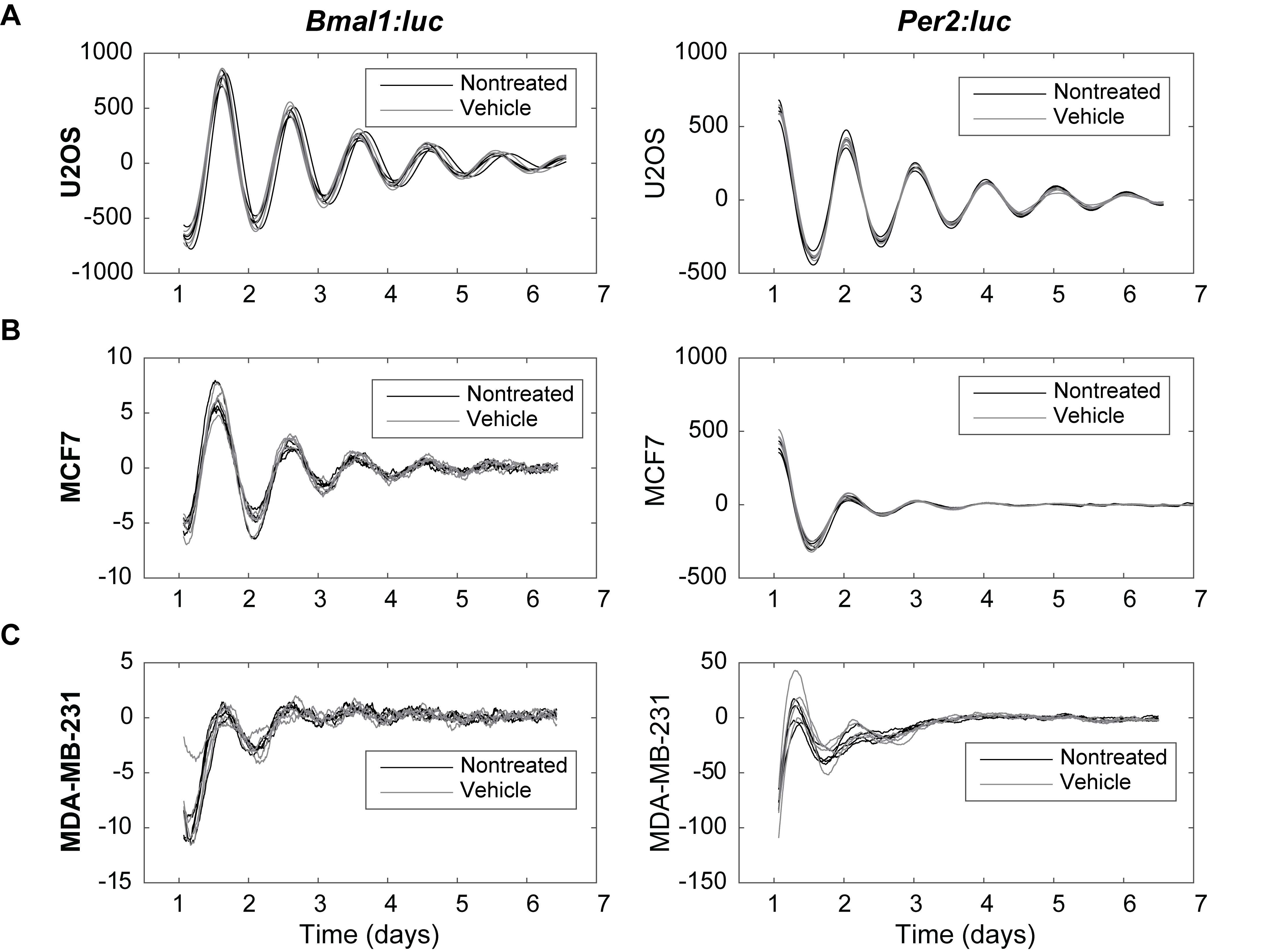

Supplement: S6 Fig — Shown are the smoothed and de-trended replicates for Bmal1:luc and Per2:luc reporters in (A) U2OS, (B) MCF7, and (C) MDA-MB-231 cells under non-treated (black) and vehicle-treated (gray) conditions. N = 4 for each treatment for each cell line. Data were evaluated beginning from t = 0.5 (day); each trace has been de-trended by subtracting the mean of a 24 h sliding window and smoothed using the mean of a 3 h sliding window, making the de-trended date begin at t = 1 (days). (TIF) [file pone.0236315.s006.tif]

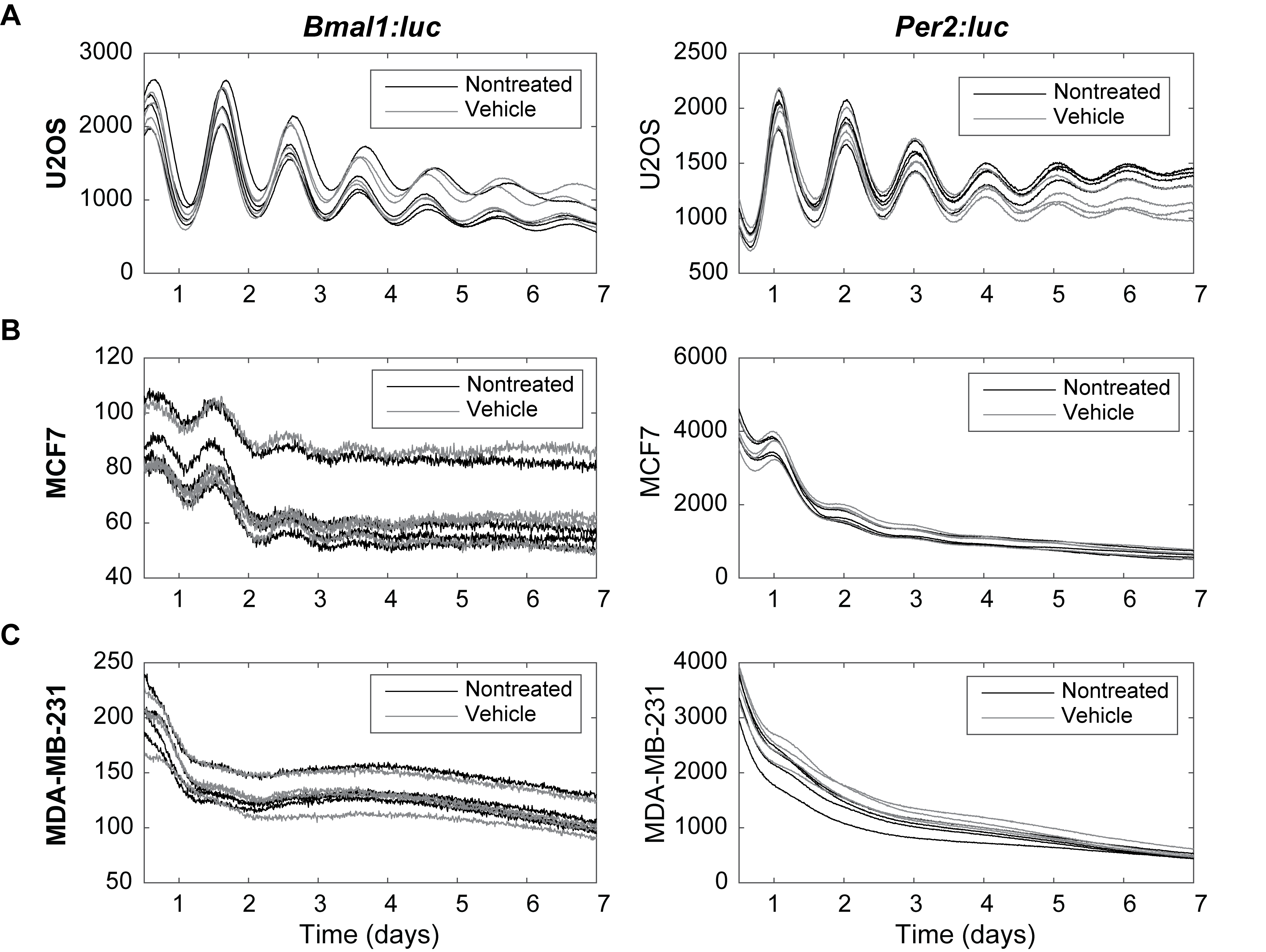

Supplement: S7 Fig — Shown are raw data replicates for Bmal1:luc and Per2:luc reporters in (A) U2OS, (B) MCF7, and (C) MDA-MB-231 cells under non-treated (black) and vehicle-treated (gray) conditions. N = 4 for each treatment for each cell line. (TIF) [file pone.0236315.s007.tif]

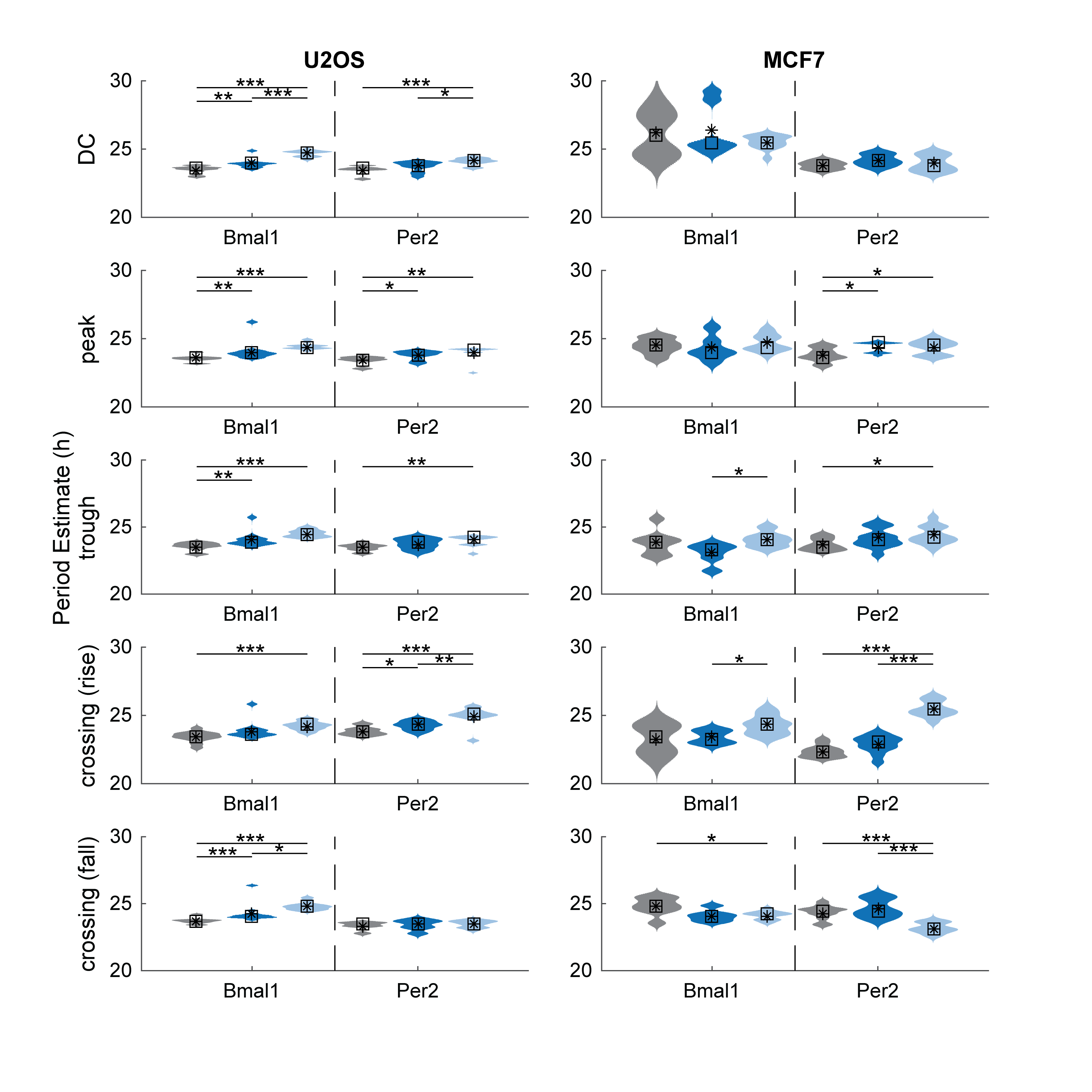

Supplement: S8 Fig — Shown are the distributions of period estimates for each recording (N = 12 for each condition for each reporter and cell line). For each cell line (U2OS on left, MCF7 on right) and each reporter (Bmal1 on left, Per2 on right), we show the period distribution, color-coded by treatment (gray = vehicle, blue = 5 μM nobiletin, light blue = 50 μM nobiletin). Each row shows results from a different method (DC = damped cosine-fitting; the remaining method names indicate the phase marker used to estimate the period, e.g. “peak” indicates the difference in time between the peak of each cycle is used). Randomization tests for difference in means were performed to determine if treatment led to statistically significant differences. P-values were corrected according to Bonferroni’s method (* p < 0.05, ** p < 0.01, *** p < 0.001). For U2OS (both reporters) and MCF7 Per2:luc, the period estimates of 50 μM nobiletin treated recordings are either longer than or not statistically significantly different from those treated with vehicle or 5 μM. For MCF7 Bmal1:luc, however, two methods (trough-to-trough estimates and mean-crossing while levels are rising) indicate that the 50 μM nobiletin treatment leads to shorter periods and one (mean-crossing while levels are falling) indicates that it leads to longer periods. (TIF) [file pone.0236315.s008.tif]

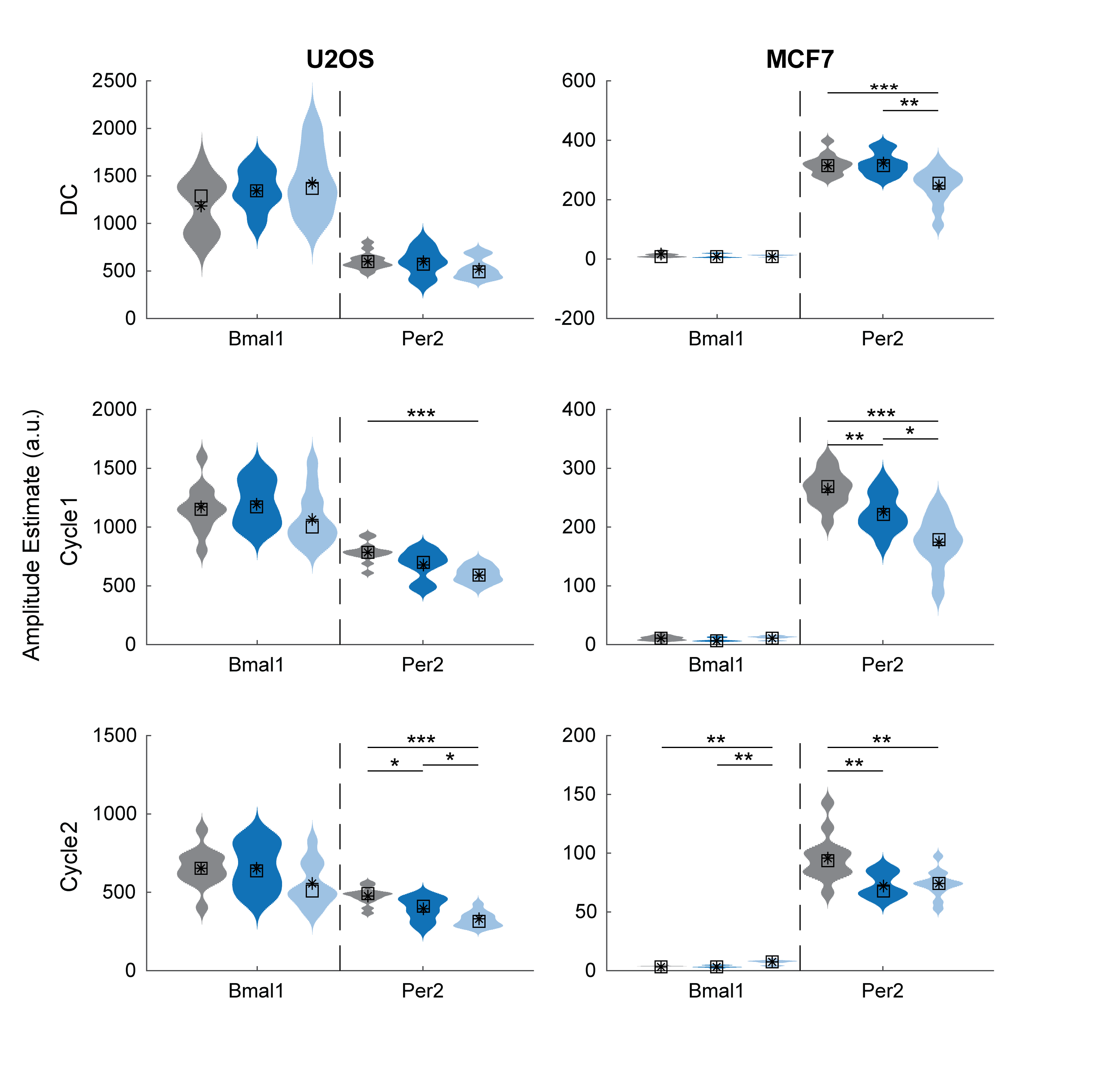

Supplement: S9 Fig — Shown are the distributions of amplitude estimates for each recording (N = 12 for each condition for each cell line). For each cell line (U2OS on left, MCF7 on right) and each reporter (Bmal1 on left, Per2 on right), we show the amplitude distribution, color-coded by treatment (gray = vehicle, blue = 5 μM nobiletin, light blue = 50 μM nobiletin). Each row shows results from a different method (DC = damped cosine-fitting; Cycle1 = difference between magnitudes of first peak and first trough; Cycle2 = difference between magnitudes of second peak and second trough). Randomization tests for difference in means were performed to determine if treatment led to statistically significant differences. P-values were corrected according to Bonferroni’s method (* p < 0.05, ** p < 0.01, *** p < 0.001). For U2OS Bmal1:luc, there are no trends. For U2OS Per2:luc, 50 μM treatment led to a smaller amplitude. For MCF7 Bmal1:luc, only the amplitude of the second cycle is different across treatments, with a 50 μM treatment leading to a higher amplitude. For MCF7 Per2:luc, 50 μM treatment led to a smaller amplitude. (TIF) [file pone.0236315.s009.tif]

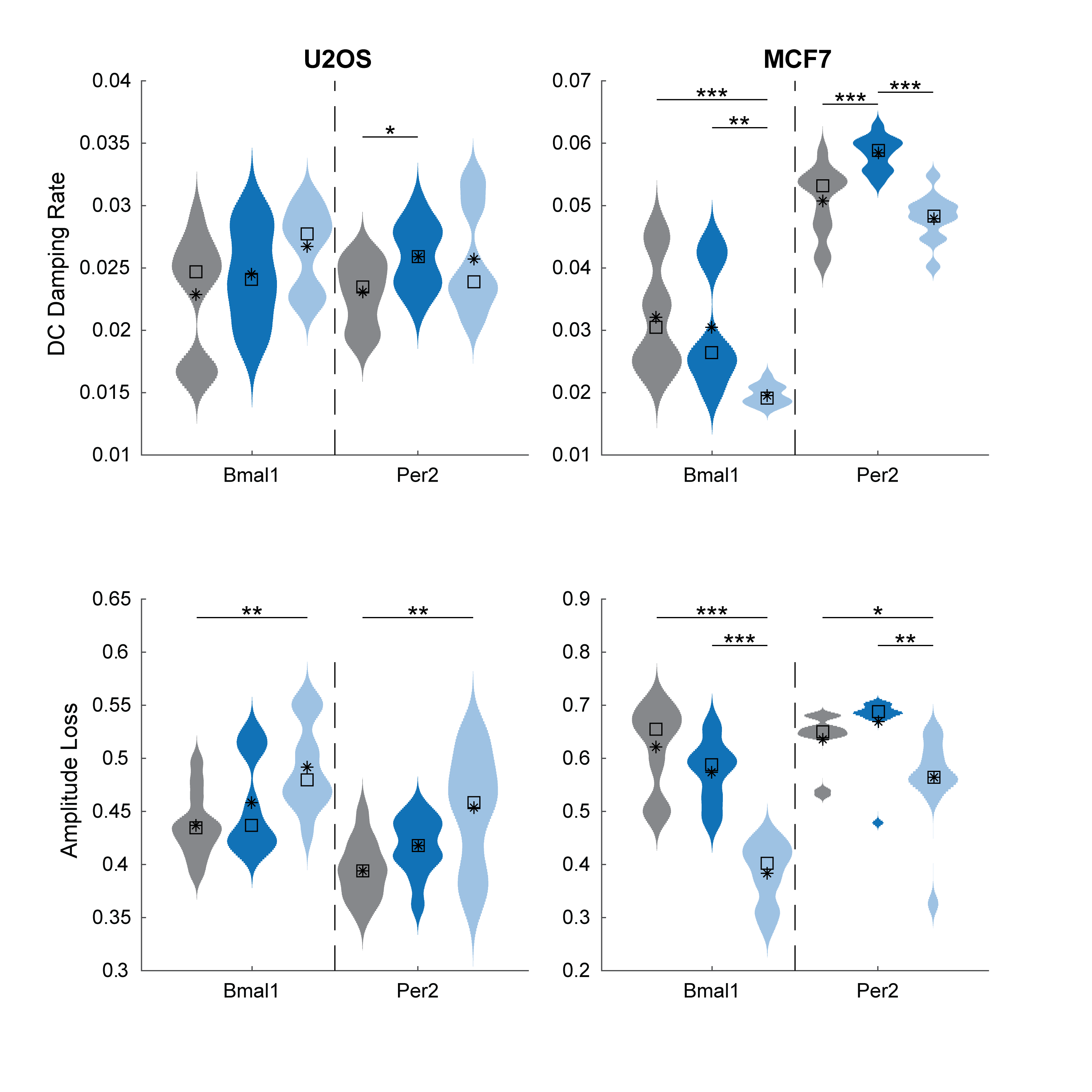

Supplement: S10 Fig — Shown are the distributions of damping estimates for each recording (N = 12 for each condition for each cell line). For each cell line (U2OS on left, MCF7 on right) and each reporter (Bmal1 on left, Per2 on right), we show the amplitude distribution, color-coded by treatment (gray = vehicle, blue = 5 μM nobiletin, light blue = 50 μM nobiletin). Each row shows results from a different method (DC Damping rate = damped cosine-fitting; Amplitude Loss = 1 –Cycle2 Amplitude/Cycle1 Amplitude). Randomization tests for difference in means were performed to determine if treatment led to statistically significant differences. P-values were corrected according to Bonferroni’s method (* p < 0.05, ** p < 0.01, *** p < 0.001). For U2OS Bmal1:luc and Per2:luc, 50 μM nobiletin treatment leads to increased amplitude loss. For MCF7, 50 μM nobiletin treatment reduces damping (as determined both by cosine-fitting and by measuring amplitude loss from cycle 1 to cycle 2). (TIF) [file pone.0236315.s010.tif]

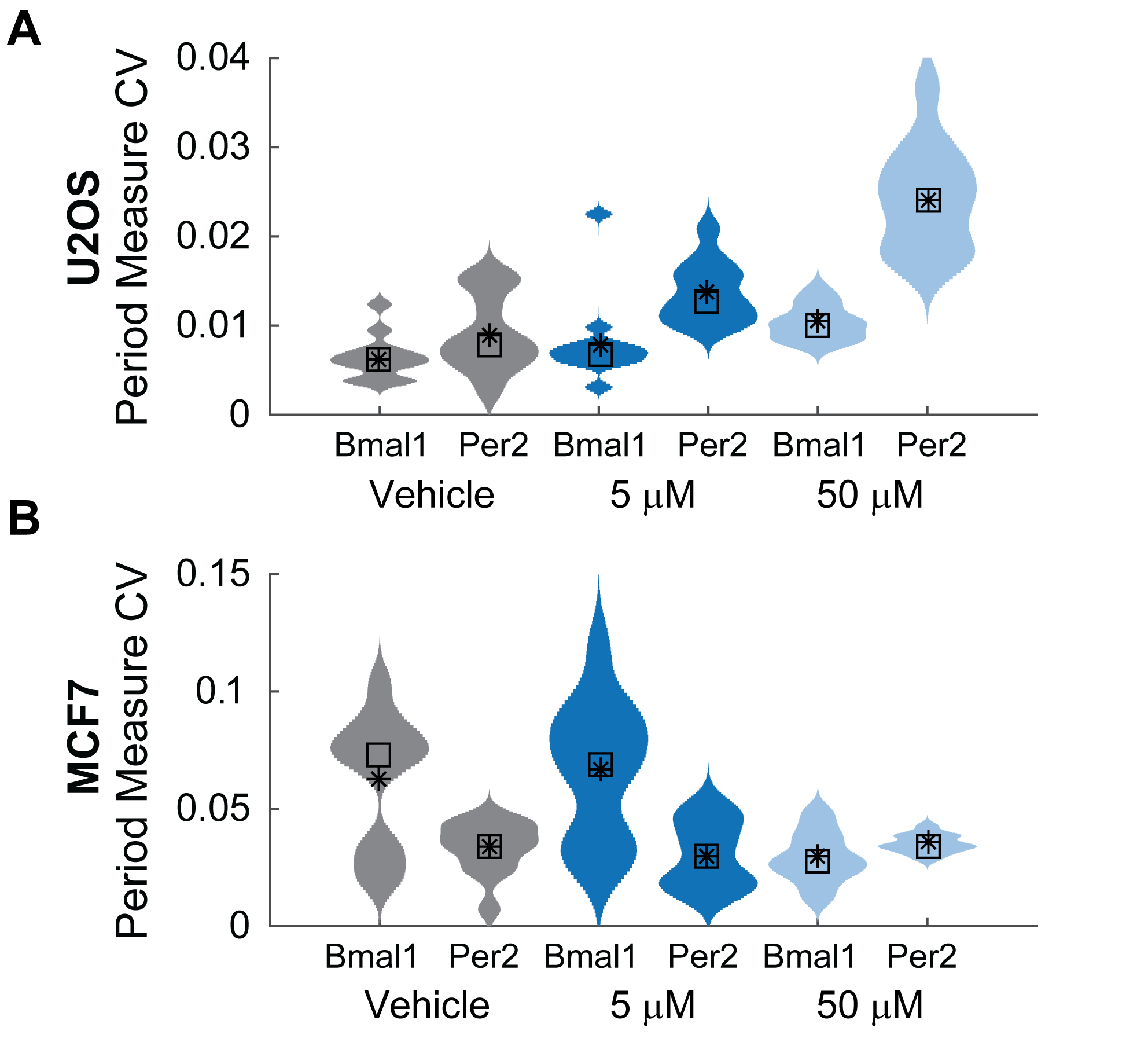

Supplement: S11 Fig — For a given reporter and treatment, each of 12 replicates had its period estimated by 5 methods (damped-sine fitting, peak-to-peak, trough-to-trough, mean-crossing-to-mean-crossing on the rise, mean-crossing-to-mean-crossing on the fall). The coefficient of variation (CV) across the 5 methods is computed. Shown are the distributions of CVs for (A) U2OS and (B) MCF7 Bmal1:luc and Per2:luc recordings. Color indicates treatment (gray = vehicle, blue = 5 μM nobiletin, light blue = 50 μM nobiletin). For U2OS, the CV of period estimation is less than 4%. For MCF7 Bmal1:luc, vehicle treatment and 5 μM nobiletin period estimates vary more (the standard deviation is 10% of the mean). (TIF) [file pone.0236315.s011.tif]

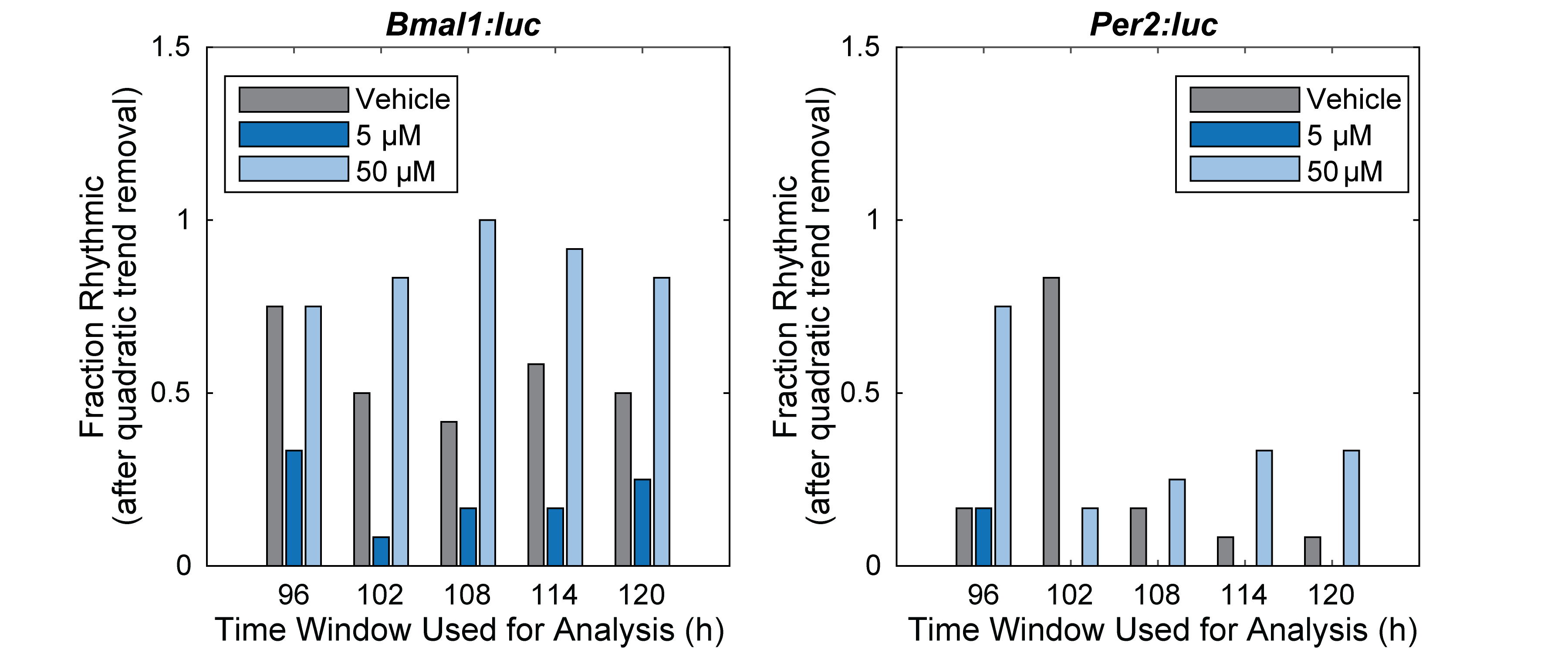

Supplement: S12 Fig — Shown are the fractions of recordings classified as rhythmic (p < 0.05 in FFT-based test), after removing a quadratic trend, using time-series with increasing end times. The fraction scoring as rhythmic depends on the extent of the time-series included in the analysis, but cells treated with 50 μM nobiletin consistently scored as rhythmic more frequently than those treated with either vehicle or 5 μM. The only exception is for Bmal1:luc recordings with the first 96 hours analyzed. (TIF) [file pone.0236315.s012.tif]

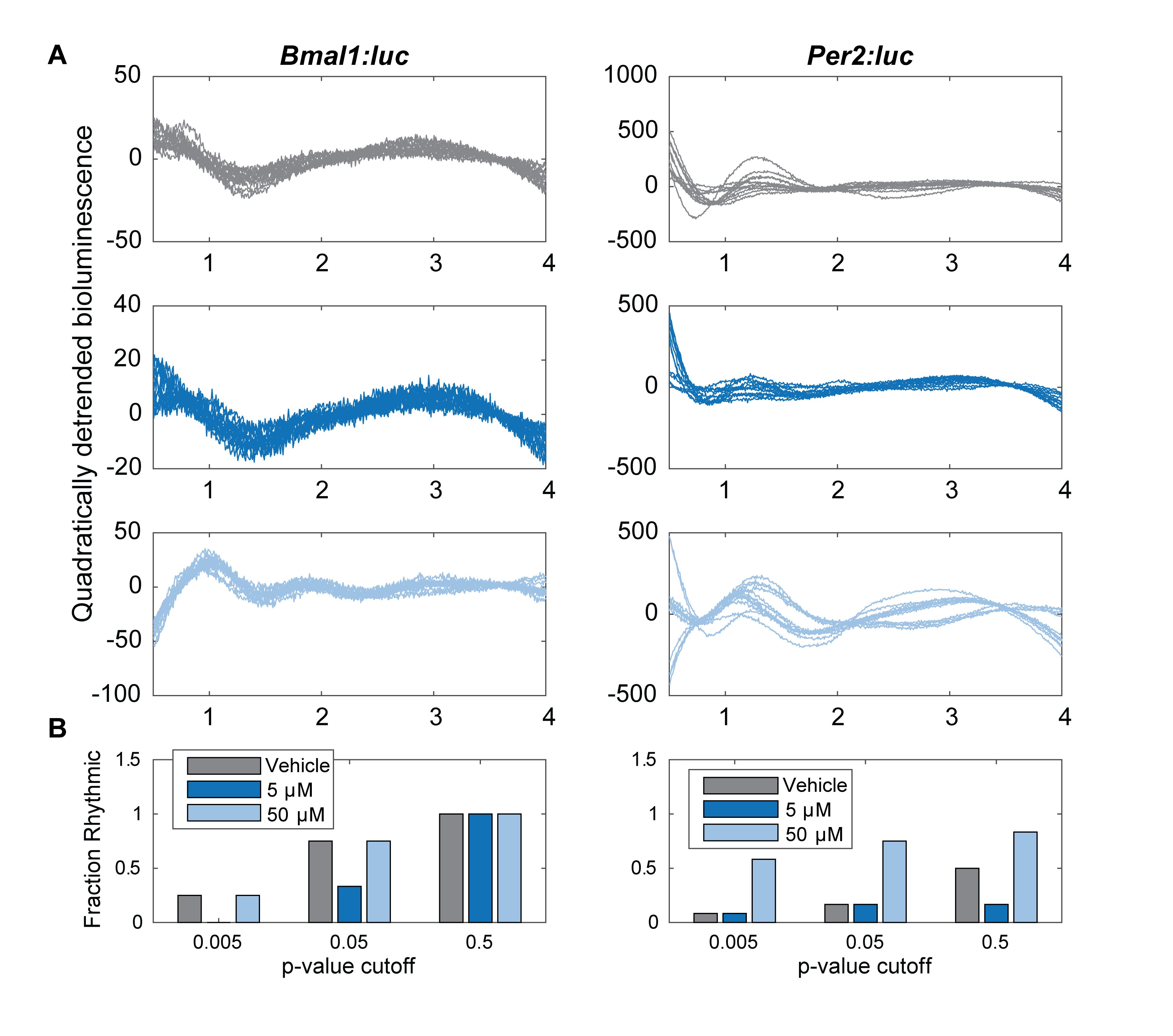

Supplement: S13 Fig — (A) Shown are luminometry recordings after removing a quadratic trend from the first 96 hours of data. Subfigure placement and color indicate treatment (top/gray = vehicle, middle/blue = 5 μM nobiletin, bottom/light blue = 50 μM nobiletin). (B) Shown are the fractions of recordings classified as rhythmic by an FFT-based test, using increasing p-value cut-offs. For Bmal1:luc, both vehicle-treated and 50 μM nobiletin-treated cells are scored as rhythmic, but visual inspection demonstrates that the rhythm is most prominent for recordings from the 50 μM nobiletin treatment. For Per2:luc, visual inspection and the fraction scored as rhythmic both indicate that rhythms are present in cells treated with 50 μM nobiletin. (TIF) [file pone.0236315.s013.tif]

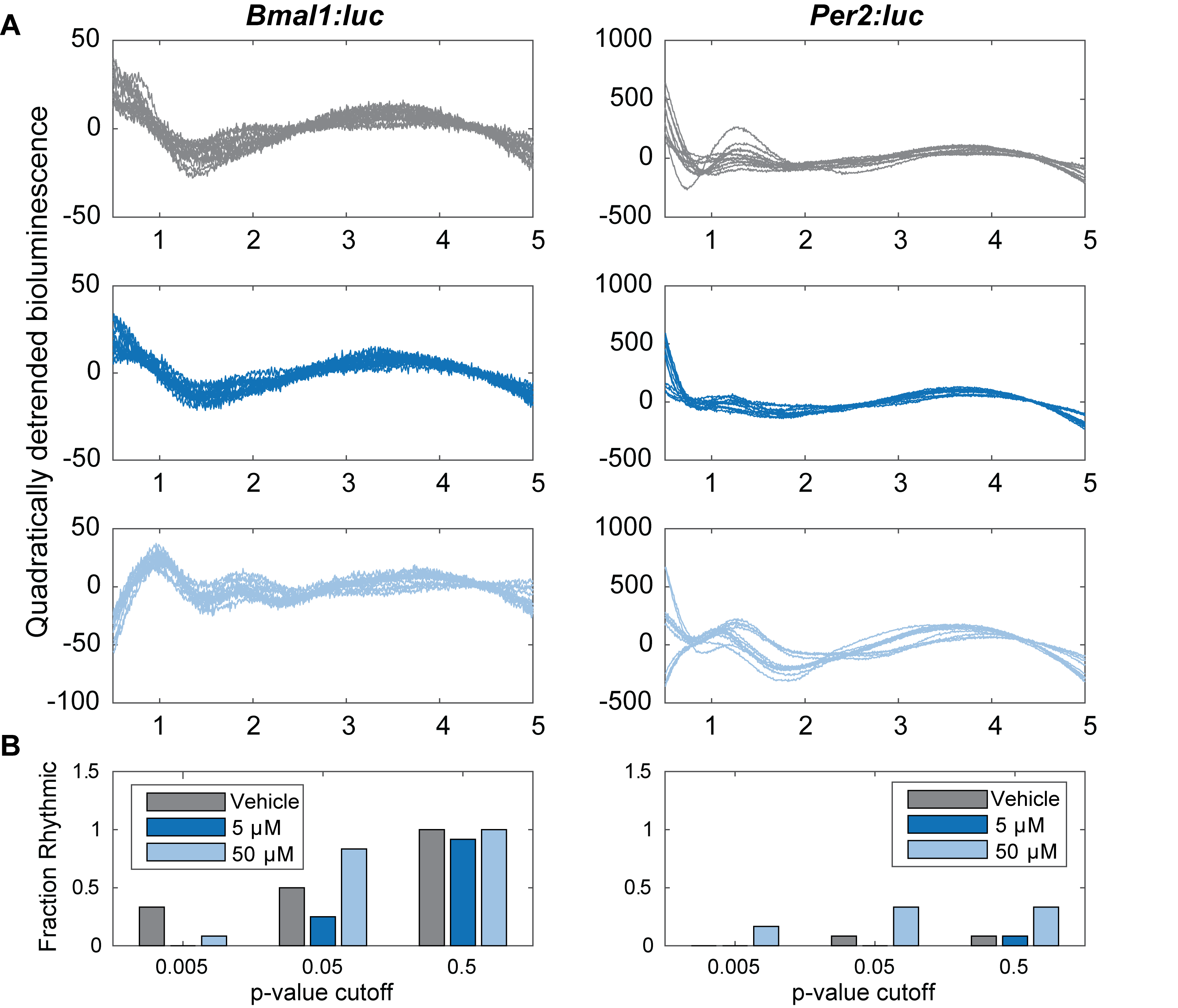

Supplement: S14 Fig — (A) Shown are luminometry recordings after removing a quadratic trend from the first 120 hours of data. Subfigure placement and color indicate treatment (top/gray = vehicle, middle/blue = 5 μM nobiletin, bottom/light blue = 50 μM nobiletin). (B) Shown are fractions of recordings classified as rhythmic by an FFT-based test, using increasing p-value cut-offs. Visual inspection and the fraction scored as rhythmic (when p = 0.05) both indicate that rhythms are present in cells treated with 50 μM nobiletin. For Per2:luc, this pattern is consistent, regardless of the size of the p-value. For Bmal1:luc, both vehicle-treated and 50 μM nobiletin-treated cells scored as rhythmic, but visual inspection demonstrates that the rhythm is most prominent for recordings from the 50 μM nobiletin treatment. For Per2:luc, visual inspection and the fraction scored as rhythmic both indicate that rhythms are present in cells treated with 50 μM nobiletin. (TIF) [file pone.0236315.s014.tif]

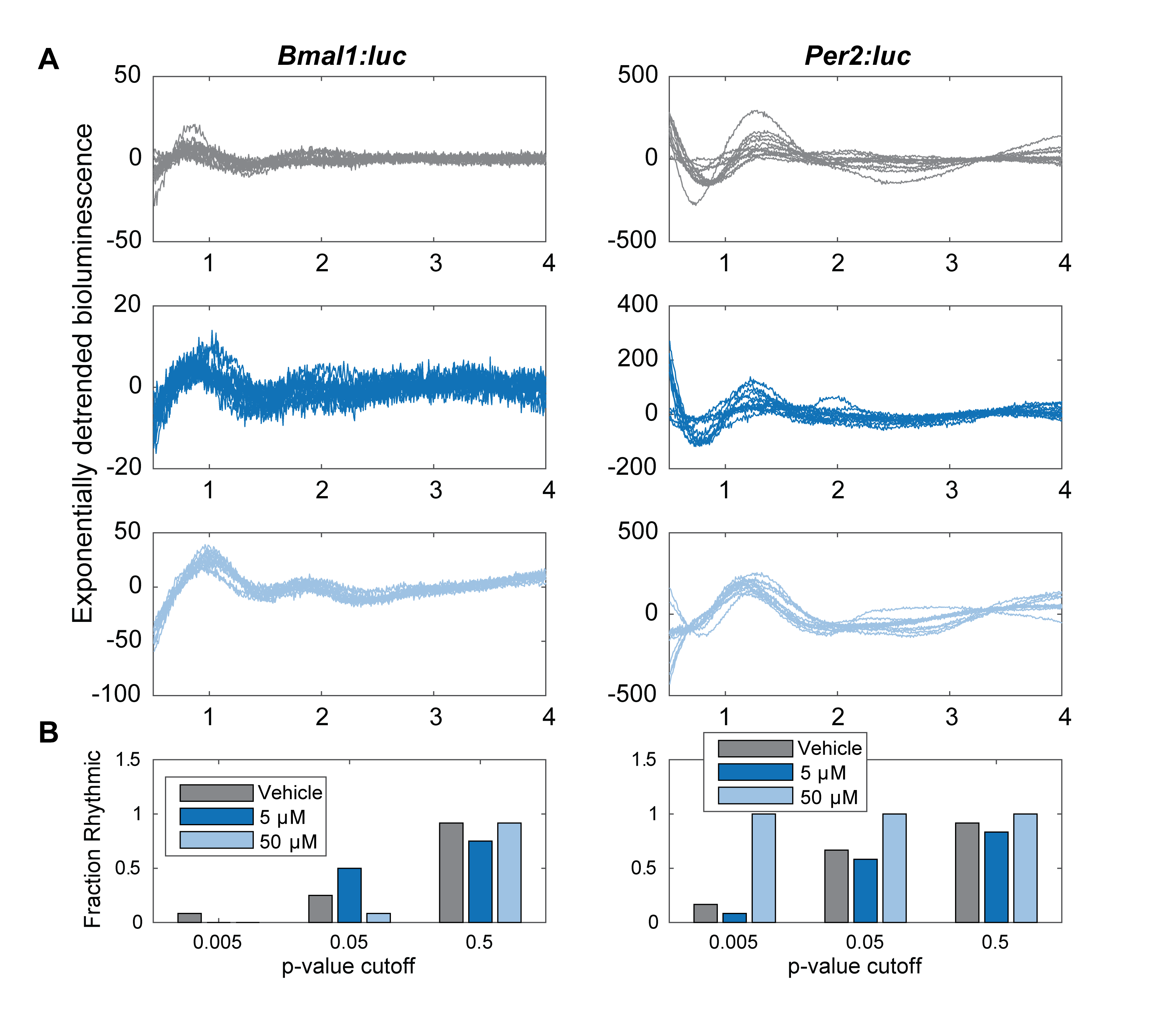

Supplement: S15 Fig — (A) Shown are luminometry recordings after removing an exponential trend from the first 96 hours of data. Subfigure placement and color indicate treatment (top/gray = vehicle, middle/blue = 5 μM nobiletin, bottom/light blue = 50 μM nobiletin). (B) Shown are fractions of recordings classified as rhythmic by an FFT-base test, using increasing p-value cut-offs. For Bmal1:luc, both vehicle- and 50 μM nobiletin-treated cells scored as rhythmic, but visual inspection demonstrates that the rhythm is most prominent for recordings from the 50 μM nobiletin treatment. For Per2:luc, visual inspection and the fraction scored as rhythmic both indicate that rhythms are present in cells treated with 50 μM nobiletin. (TIF) [file pone.0236315.s015.tif]

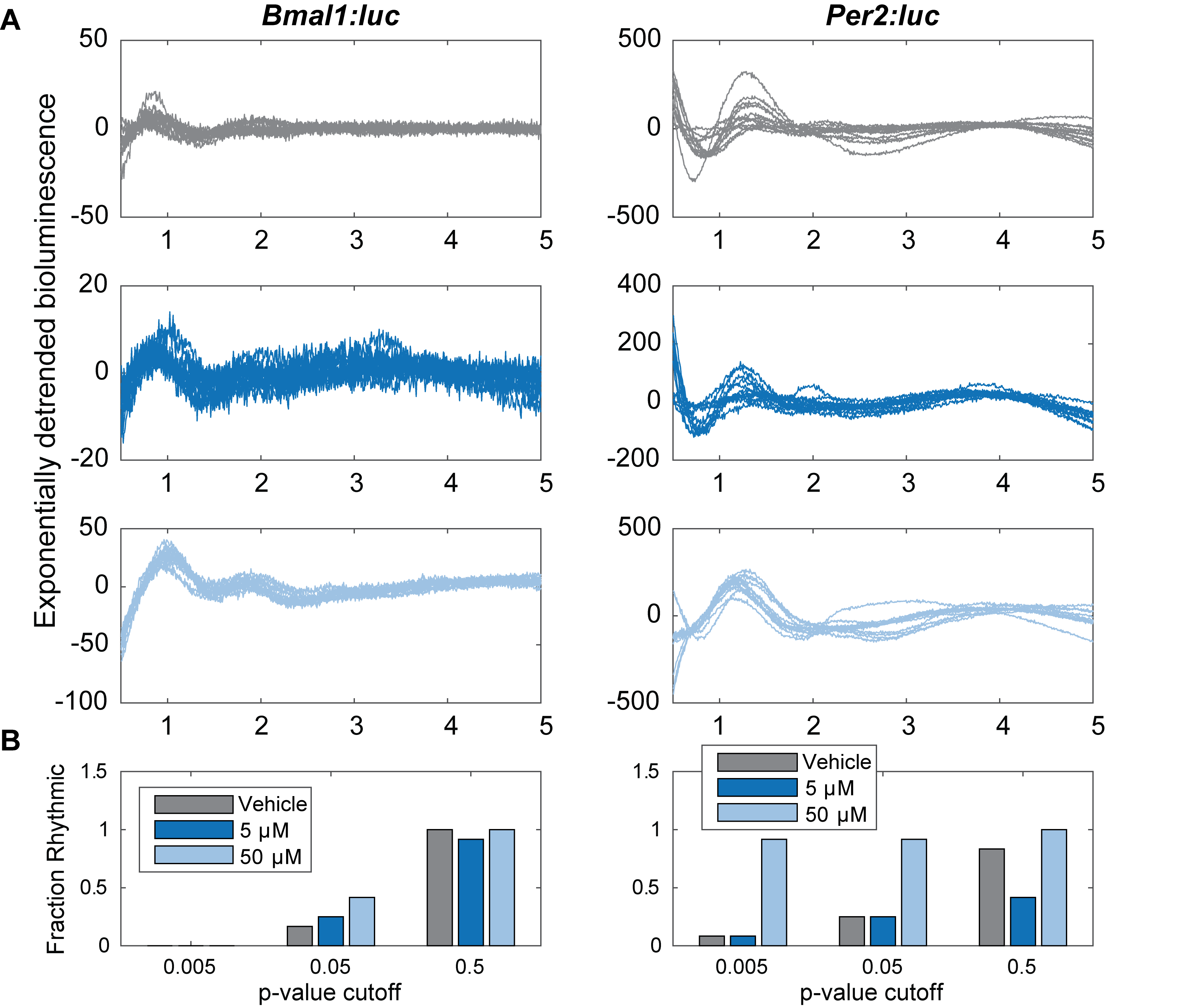

Supplement: S16 Fig — (A) Shown are luminometry recordings after removing an exponential trend from the first 120 hours of data. Subfigure placement and color indicate treatment (top/gray = vehicle, middle/blue = 5 μM nobiletin, bottom/light blue = 50 μM nobiletin). (B) Shown are the fractions of recordings classified as rhythmic by and FFT-base test, using increasing p-value cut-offs. Visual inspection and the fraction scored as rhythmic (when p = 0.05) both indicate that rhythms are present in cells treated with 50 μM nobiletin. For Per2:luc, this pattern is consistent, regardless of the size of the p-value. (TIF) [file pone.0236315.s016.tif]

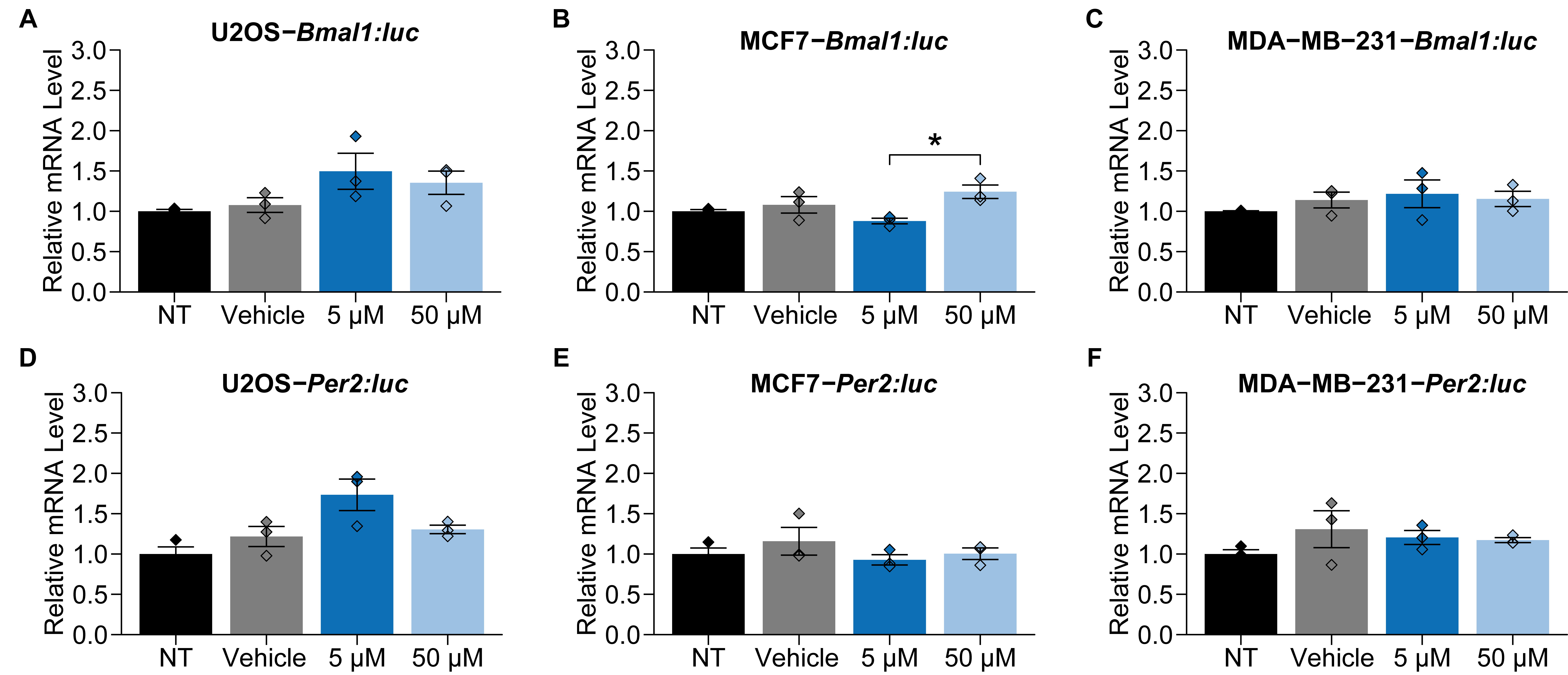

Supplement: S17 Fig — mRNA levels were quantified using RT-PCR. No significant differences were observed in nobiletin treated samples compared to vehicle treated samples in any of the cell lines tested. Each treatment contained three biological replicates, with three technical replicates each. Error bars represent SEM. Statistical significance was evaluated via two tailed t-test in R ggpubr library under equal variance, and 0.95 confidence interval (* p < 0.05). NT = non-treated; Vehicle = 0.2% DMSO. (TIF) [file pone.0236315.s017.tif]

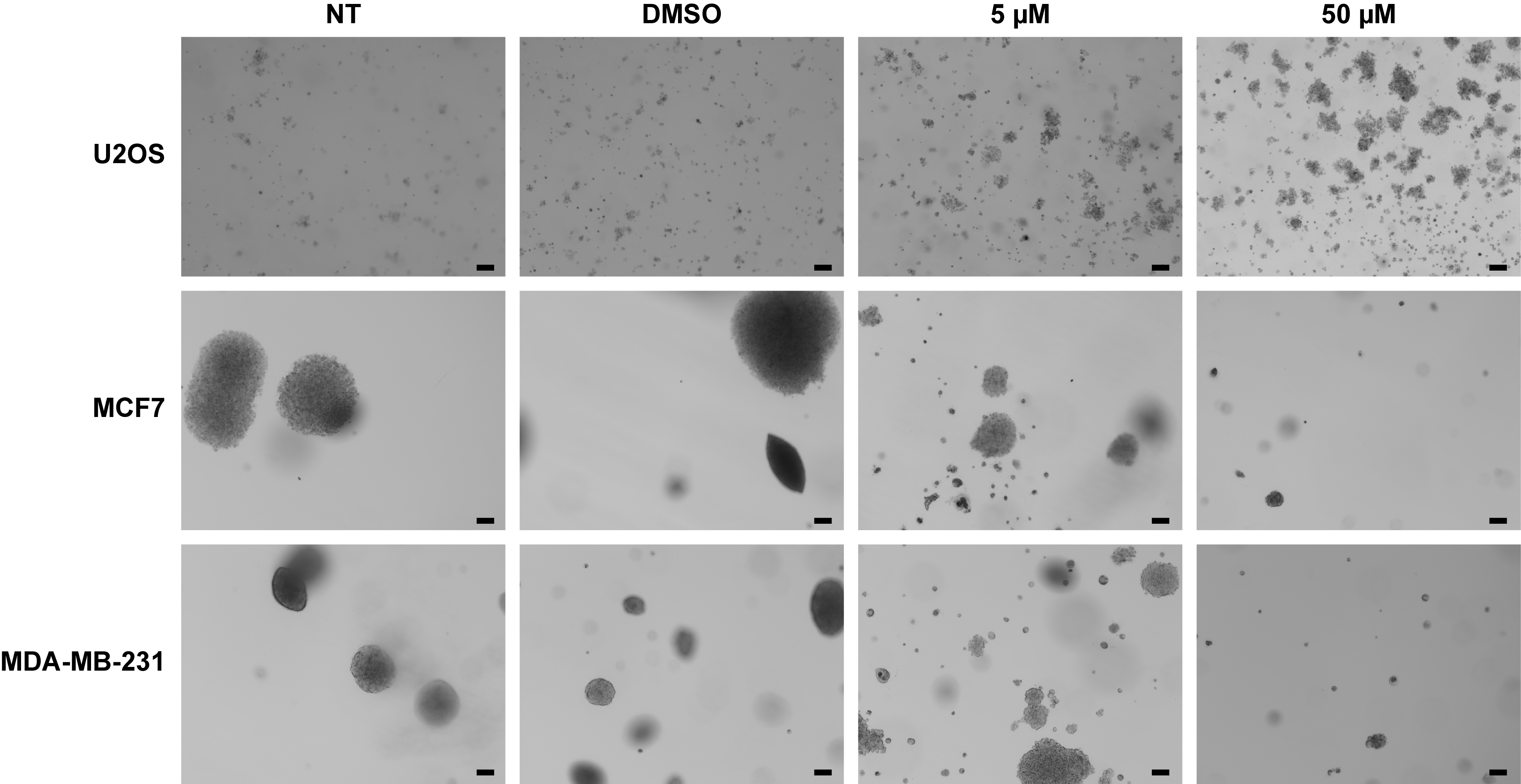

Supplement: S18 Fig — A single image for each treatment and cell line is shown. Scale bars are 106 μm. (TIF) [file pone.0236315.s018.tif]
